# Supplementary material for: Gradient tantalum-doped hematite homojunction photoanode improves both photocurrents and turn-on voltage for solar water splitting
Source: Nat Commun. 2020 Sep 15;11:4622. doi: 10.1038/s41467-020-18484-8 (PMC7493915; doi:10.1038/s41467-020-18484-8)
Supplement: Supplementary file 1 — Supplementary Information [file 41467_2020_18484_MOESM1_ESM.pdf]

Supplementary Information

Gradient tantalum-doped hematite homojunction photoanode improves both photocurrents and turn-on voltage for solar water splitting

By Zhang et al.

Supplementary Figures

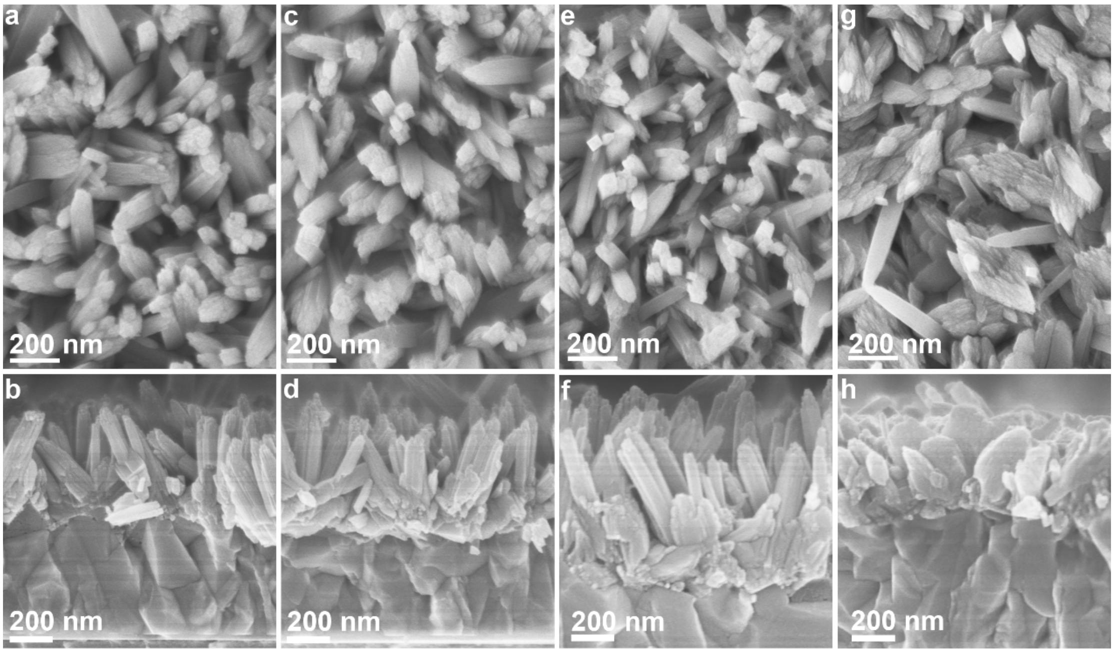

Supplementary Fig. 1| Morphology of Ta:FeOOH nanorods with different Ta volumes: (**a**, **b**) 75 μL, (**c**, **d**) 150 μL, (**e**, **f**) 300 μL, (**g**, **h**) 600 μL. The nanorod morphology of Ta:FeOOH remains similar with Ta volume of 75-300 μL. Then it changes into big nanoparticles above 600 μL. The volume of 300 μL was selected as the optimum doping solution.

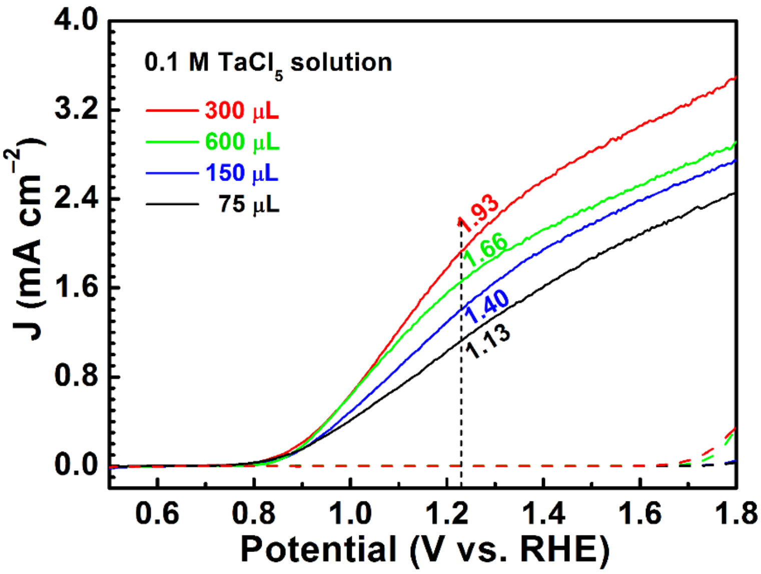

Supplementary Fig. 2| ***J–V*** **curves of the synthesized photoelectrodes with different Ta volumes.** With the increase of Ta volume up to 300 μL, the corresponding photocurrent increases gradually. At 600 μL, however, the photocurrent is lower than that of 300 μL.

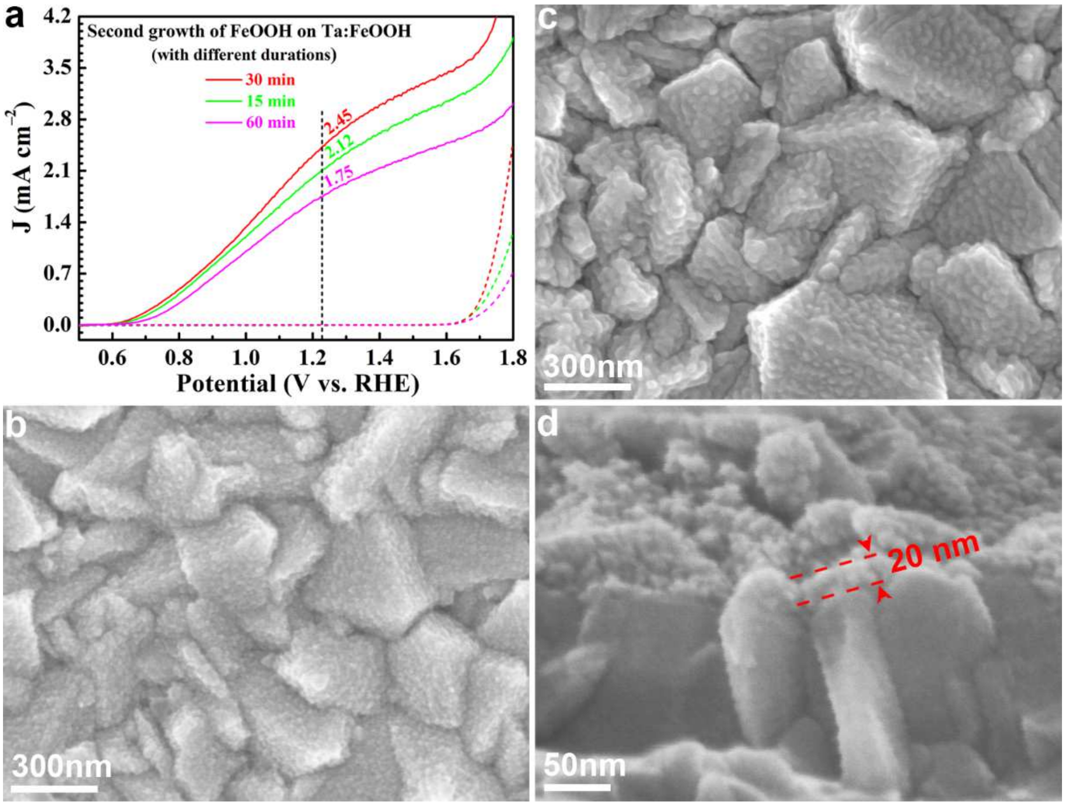

Supplementary Fig. 3| The second hydrothermal growth of a thin FeOOH layer on Ta:FeOOH with different durations. (**a**) *J–V* curves of corresponding photoelectrodes with different durations. (**b**) Hydrothermal growth of thin layer FeOOH for 30 min on bare FTO. (**c**) Corresponding morphology and (**d**) cross-section image after HMA treatment. The layer thickness is about 20 nm for 30 min hydrothermal growth estimated from the cross-section image in d.

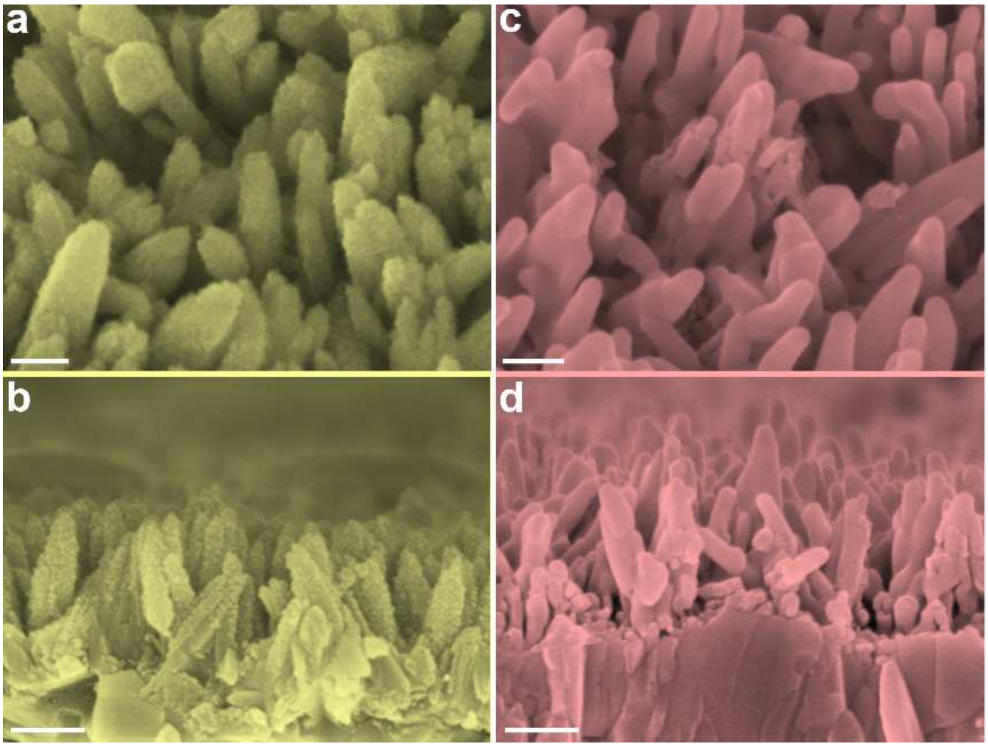

Supplementary Fig. 4| Morphologies of homojunction nanorods. SEM images of Ta:FeOOH@FeOOH (**a**, **b**) and Ta:Fe_2O_3@Fe_2O_3 (**c**, **d**). The scale bar in (**a**, **c**) and (**b**, **d**) is 100 and 200 nm, respectively.

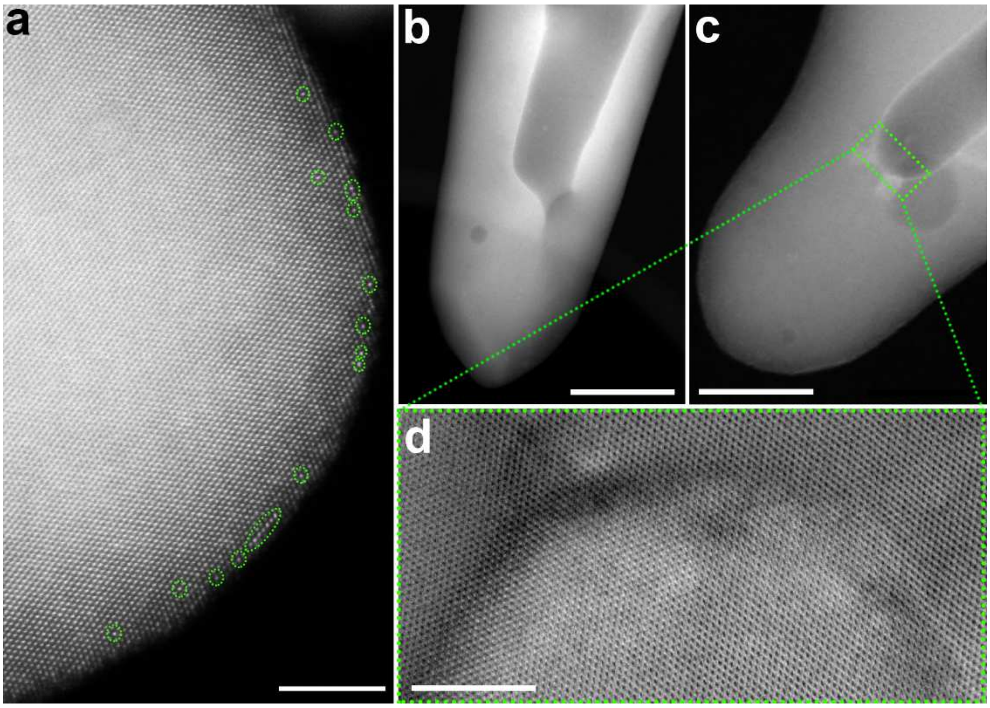

Supplementary Fig. 5| STEM images for Ta:Fe_2O_3 and Ta:Fe_2O_3@Fe_2O_3. (**a**) HRSTEM image of Ta:Fe_2O_3 nanorods (circles denote Ta atoms). (**b**, **c**) STEM images of Ta:Fe_2O_3@Fe_2O_3 homojunction nanorods. (**d**) HRSTEM of homojunction nanorod. The scale bar is 5 nm in (**a**, **d**), 50 nm in (**b**) and 30 nm in (**c**).

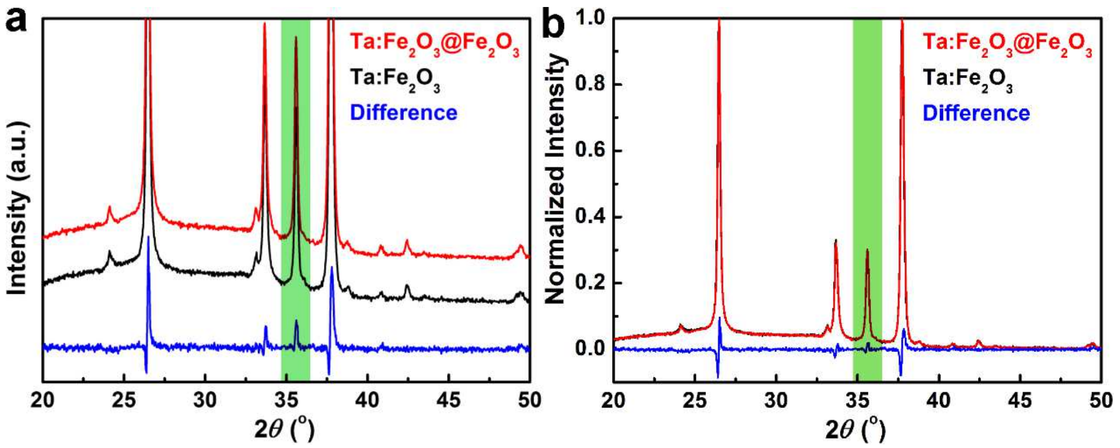

Supplementary Fig. 6| Difference of pristine (a) and normalized XRD (b) of Ta:Fe_2O_3@Fe_2O_3 and Ta:Fe_2O_3. Green area denotes (110) peak. Clearly, the difference spectrum of original XRD patterns (**a**) show a little difference of (110) peak and virtually none of (102) and (104) peaks, which indicates the highly preferential orientation of (110) even under second hydrothermal growth. However, the difference spectrum by normalized XRD patterns (**b**) shows a negligible difference.

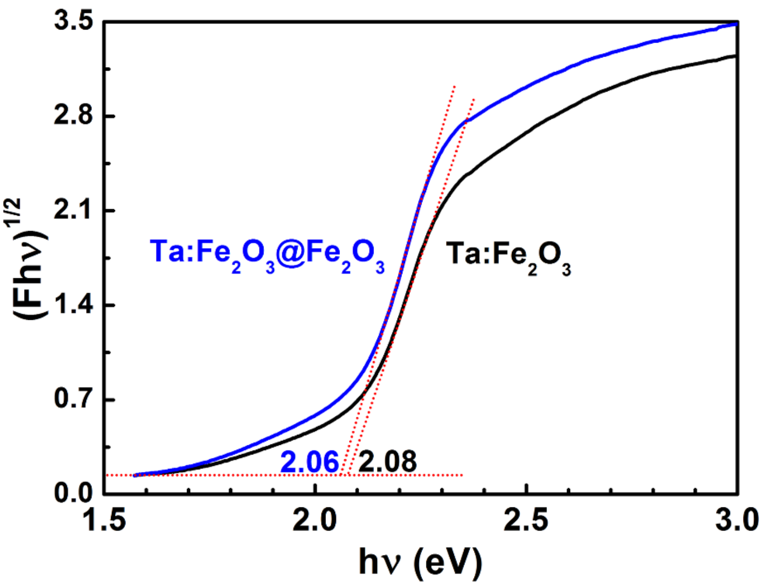

Supplementary Fig. 7| Band gaps determined from light absorption spectra. The band gap estimated from the respective light absorption is 2.08 and 2.06 eV for Ta:Fe_2O_3 and Ta:Fe_2O_3@Fe_2O_3 photoanodes, which are close to the reported value of ~2.1 eV.

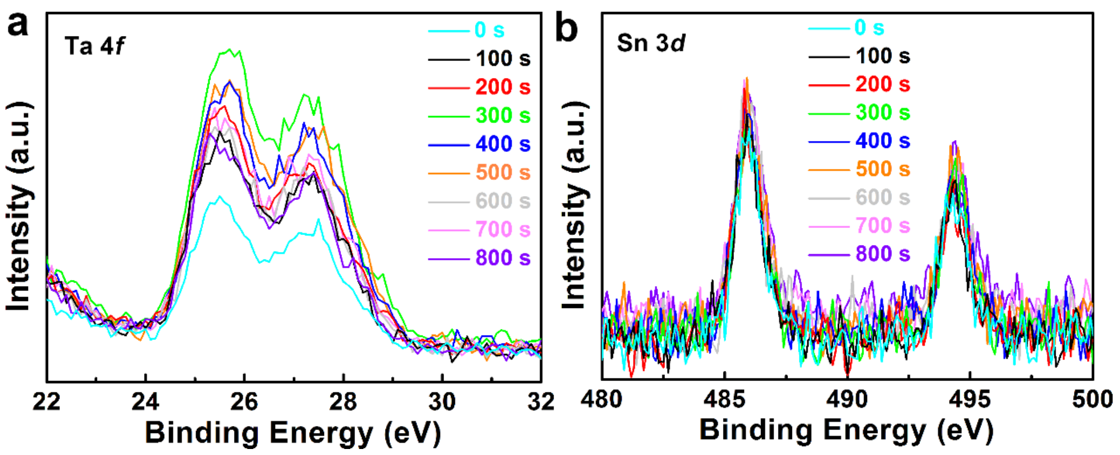

Supplementary Fig. 8| XPS depth profiles of Ta:Fe_2O_3@Fe_2O_3 homojunction nanorods. (a) Ta 4*f*. (b) Sn 3*d*. With the increase of etching time, the intensity of Ta 4*f* signal firstly becomes strong and then decreases gradually (**a**), indicating that there exists an interface of Ta concentration, which further evidences the formation of homojunction. In contrast, the Sn 3*d* signal maintains a gradual increase during the whole etching process (**b**). There is not a clear interface of Sn concentration owing to the significant suppression of Sn diffusion by in-situ doped Ta atoms in FeOOH nanorods.

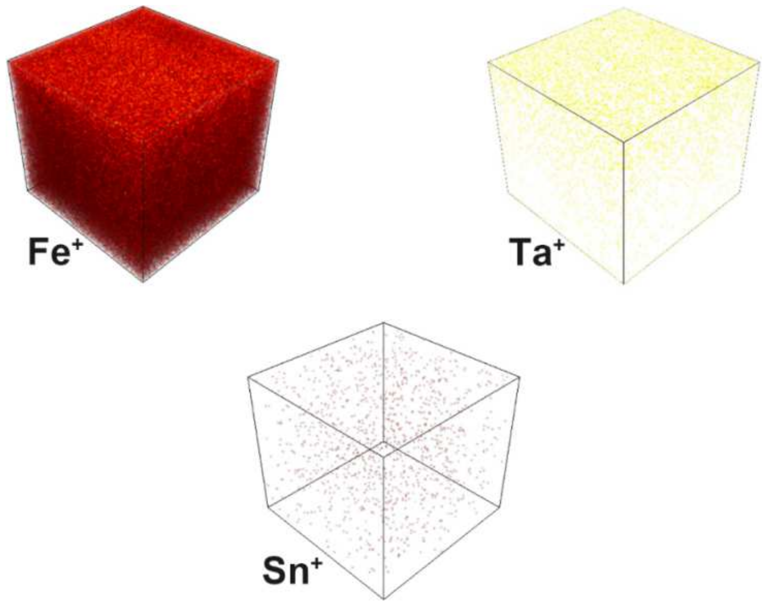

Supplementary Fig. 9| Three-dimensional (3D) visualization of SIMS profiles of homojunction. Both of Fe^+ and Sn^+ signals are homogeneously distributed. However, Ta^+ signal clearly shows a gradient concentration from top to bottom.

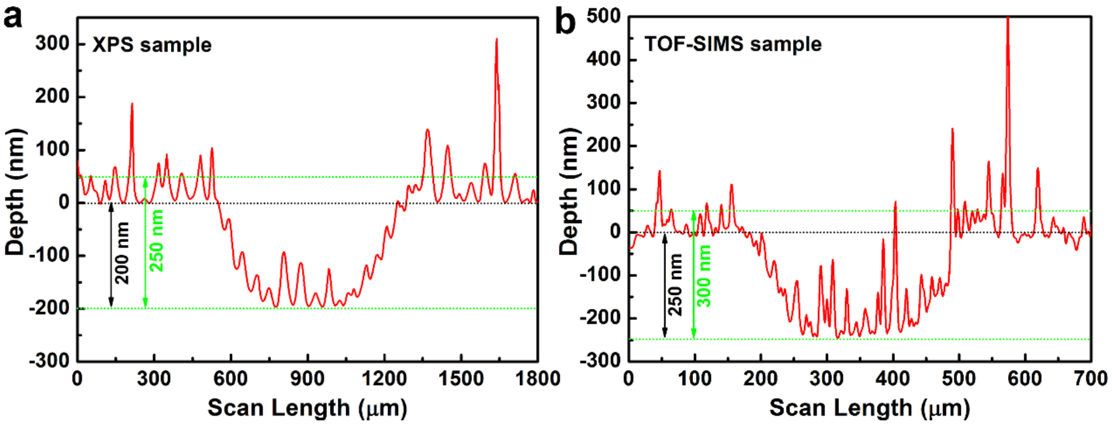

Supplementary Fig. 10 Surface profiler profiles of XPS (a) and TOF-SIMS (b) samples. The etched depths of XPS and TOF-SIMS samples are estimated to be 200~250 nm and 250~300 nm, which corresponds to the etching rates of 6~7.5 nm/min and 7.5~9 nm/min, respectively.

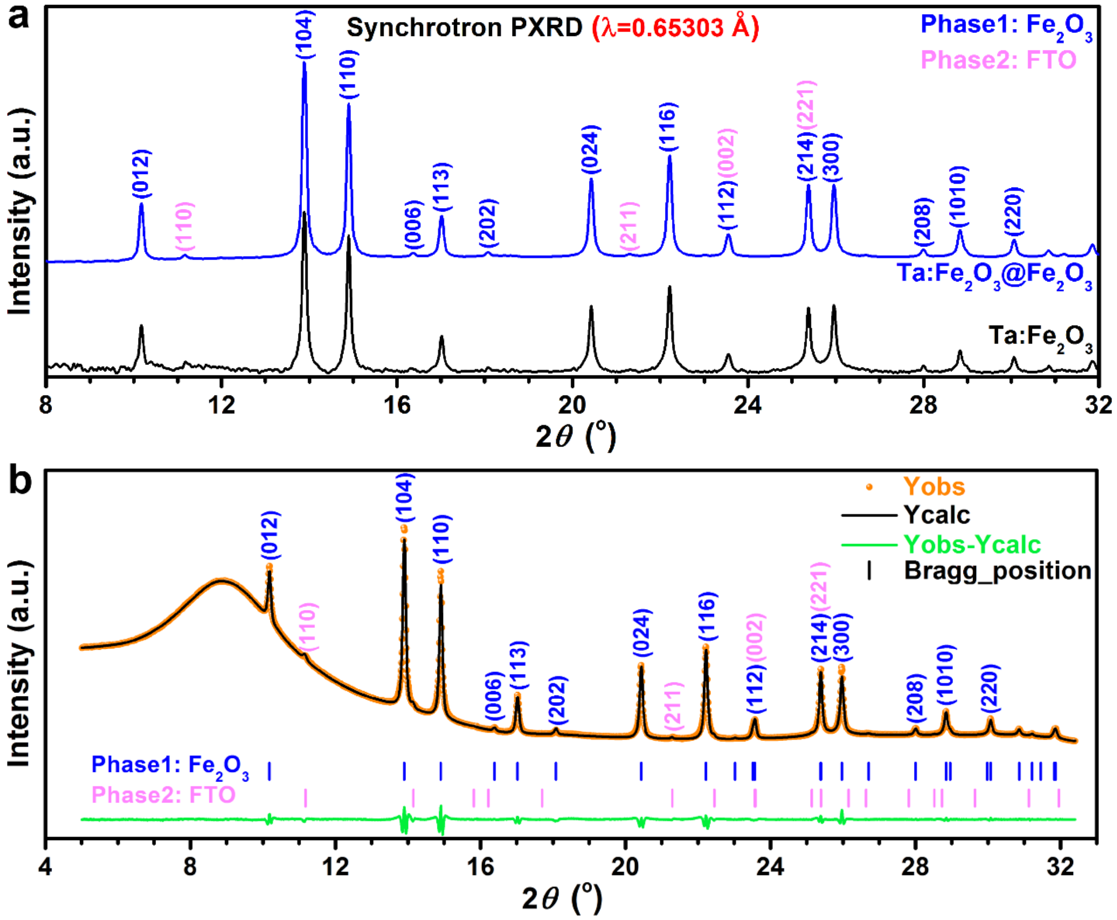

c

	Fe_2O_3	FTO
**Space group**	R-3 c	P 42/m n m
**Cell parameters**	a = b = 5.0342(6) Å, c = 13.747(2) Å	a = b = 4.744(1) Å , c = 3.196(2) Å
	α = β = γ = 90°	α = β = 90°, γ = 120°
**Reliability factors**	R_p: 0.592%, R_{wp}: 1.31%, R_{exp}: 9.16%, S: 0.14301	

Supplementary Fig. 11| Synchrotron PXRD (λ=0.65303 Å). Synchrotron PXRD patterns of Ta:Fe_2O_3 and Ta:Fe_2O_3@Fe_2O_3 (**a**). Rietveld refinement profile of Ta:Fe_2O_3@Fe_2O_3 by the Full Prof Suite software (**b**). Parameter table of the Rietveld refinement (**c**). Here, Yobs, Ycalc, Yobs-Ycalc, Bragg_position, R_p, R_{wp}, R_{exp}, S represent the experimental data, the calculated data, the difference of experimental and calculated data, Bragg's position, the profile factor, the weighted profile R factor, the expected R factor, goodness of fit, respectively. Note that lattice parameters, isotropic strain broadening, asymmetry, isotropic size broadening, and GauSiz are considered for the reliable profile matching. Note that lattice parameters, isotropic strain broadening, asymmetry, isotropic size broadening, and GauSiz are considered for the reliable profile matching.

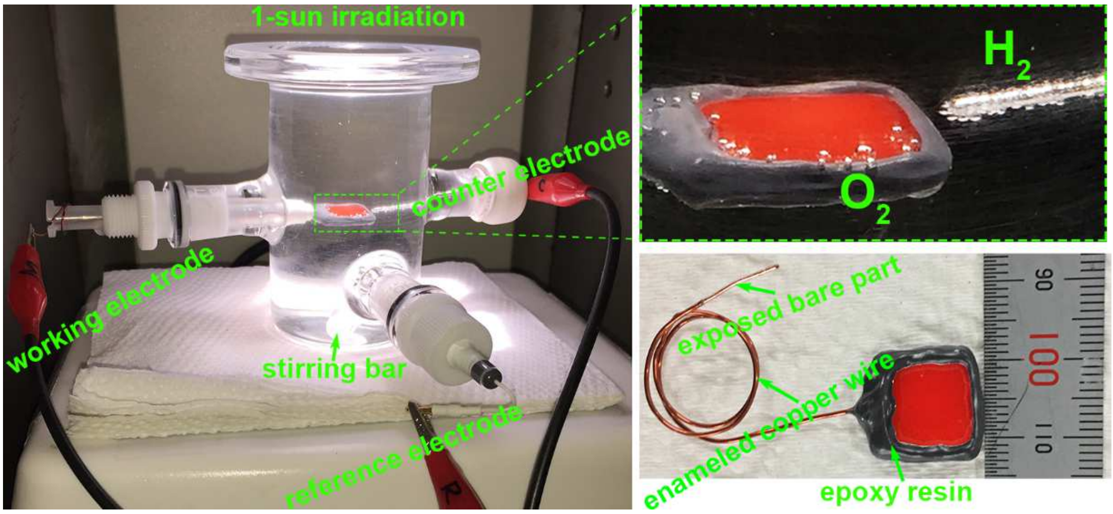

Supplementary Fig. 12| A typical PEC system used in this study.

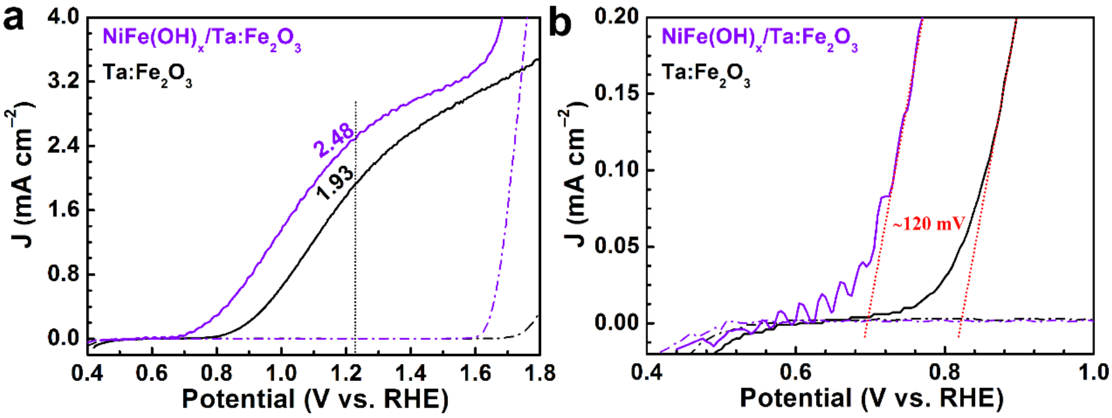

Supplementary Fig. 13| (a) *J–V* curves of Ta:Fe_2O_3 with and without NiFe$(OH)_x$ cocatalyst, and (b) the extracted V_{on} shift. The cocatalyst NiFe$(OH)_x$ provides a significant effect on Ta:Fe_2O_3 photoanode, reducing V_{on} by ~120 mV and increasing J_{ph} by 28.5 % (from 1.93 to 2.48 mA cm^{-2} at 1.23 V_{RHE}).

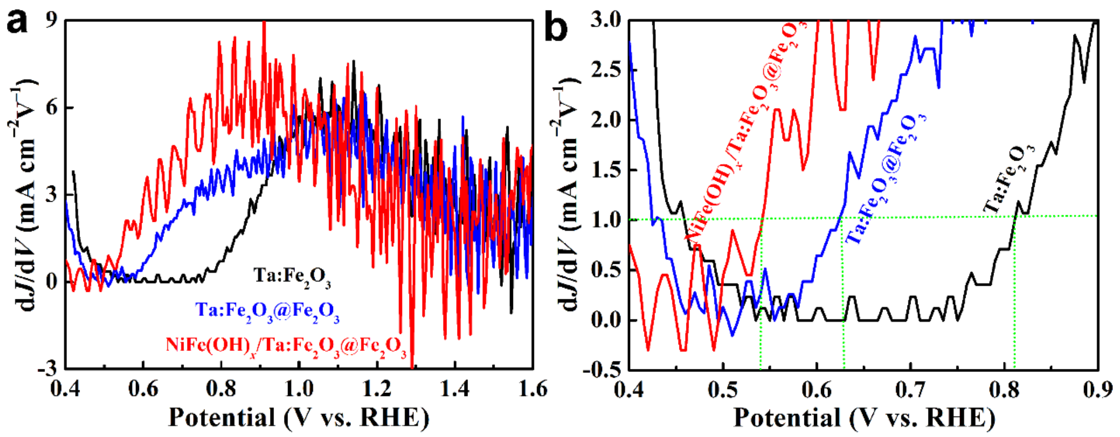

Supplementary Fig. 14| (a) Evaluated V_{on} by first-order derivative of the corresponding J–V curves. (b) Enlarged part of (a). In order to have a consistent comparison, the first-order derivative method was developed by Grätzel group,[1] which is defined as the value, at which dJ/dV > 0.2 mA cm^{-2}V^{-1} from the obtained J–V curves. The method was adopted and confirmed reasonable by Wang[2] and Ye[3] groups. When photoanodes have a high J (over 3 mA cm^{-2} at 1.23 V_{RHE}) and/or a strong signal noise, however, it is difficult to apply the criterion of dJ/dV > 0.2 mA cm^{-2}V^{-1}. In our case (**a** and **b**), it might be more reasonable to evaluate V_{on} at which dJ/dV > 1 mA cm^{-2}V^{-1} (just above the strong noise of all samples), which gives V_{on} of 0.81 (Ta:Fe_2O_3), 0.63 (Ta:Fe_2O_3@Fe_2O_3) and 0.54 V_{RHE} (NiFe(OH)$_x$/Ta:Fe_2O_3@Fe_2O_3), respectively. The results are almost the same as our extracted values (Fig. 5b) of 0.82, 0.63 and 0.55 V_{RHE}, respectively, by extrapolation-interception in a certain range of J (0 to 0.2 mA cm^{-2}) and V (0.4 to 1.0 V_{RHE}). The reasonability is also confirmed in our previous report.[4]

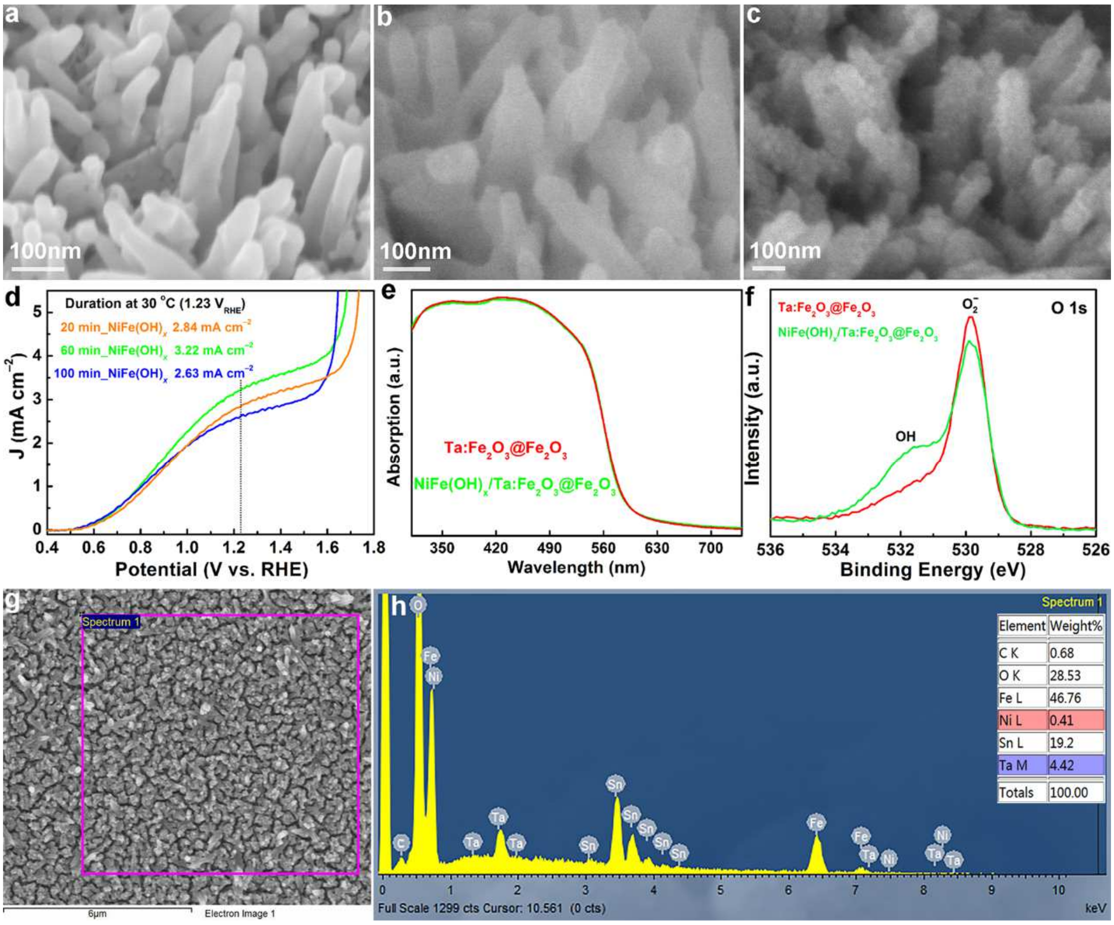

Supplementary Fig. 15| Optimization and characterization of NiFe(OH)$_x$ cocatalyst. SEM images (**a**, 20 min. **b**, 60 min. **c**, 100 min.) and corresponding *J*–*V* curves (**d**) with different durations at 30 ^{o}C. Light absorption before and after optimal (60 min duration) cocatalyst modification (**e**). The nickel and iron chloride solution can be gradually hydrolyzed to form NiFe(OH)$_x$ in a dilute concentration. From SEM images (barely taken without any sputtering of noble metal), 20 min duration (**a**) gives an invisible deposition, but shows an improved J_{ph} (2.84 mA cm^{-2}). With 60 min duration, however, a thin and uniform layer of NiFe(OH)$_x$ was deposited (**b**) because some small nanoparticles can be seen. Besides, the poor contrast of image relative to the previous one also indicates the successful deposition of NiFe(OH)$_x$, providing a maximum of J_{ph} (3.22 mA cm^{-2}). On the other hand, this thin and uniform NiFe(OH)$_x$ layer does not change any light absorption (**e**). When duration reaches 100 min, a great number of bigger nanoparticles can be observed clearly(**c**), which actually decreases the J_{ph} (2.63 mA cm^{-2}) due to too much an amount. XPS O 1s spectra (**f**) shows that the OH peak intenensity of homojunction modified with cocatalyst becomes clearly higher than that of bare one. Besides, SEM-EDS (**g, h**) detects the signal of Ni element (~0.41wt%). Our previous results demonstrated that this strategy deposited FeOOH cocatalyst on hematite successfully. Based on these results, it is reasonable that the cocatalyst is NiFe(OH)$_x$.

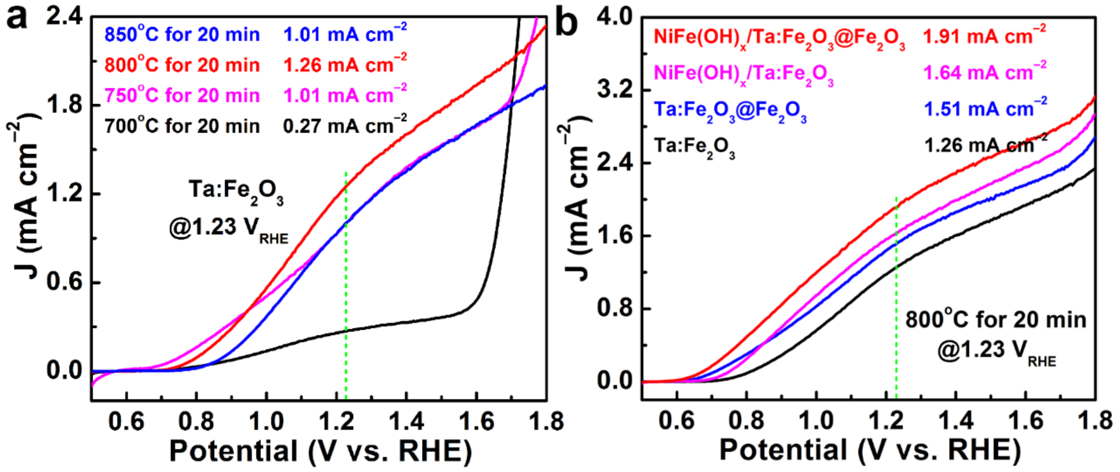

Supplementary Fig. 16| *J–V* curves of the photoelectrodes prepared by CTA. From *J–V* curves (a), the optimization condition for Ta:Fe_2O_3 photoanode is 800°C for 20 min. Higher or lower temperature would obviously decrease the photocurrent density. CTA homojunction shows an enhanced J_{ph} and a cathodic shift of V_{on} relative to CTA Ta:Fe_2O_3 (b), which indicates the existence of built-in electric field in CTA homojunction as well (Further study is needed to confirm whether it is also a gradient homojunction). However, all the photoelectrodes by CTA with/without NiFe(OH)$_x$ cocatalyst (1.26, 1.51, 1.64, and 1.91 mA cm^{-2} for Ta:Fe_2O_3, Ta:Fe_2O_3@Fe_2O_3, NiFe(OH)$_x$/Ta:Fe_2O_3, NiFe(OH)$_x$/Ta:Fe_2O_3@Fe_2O_3, respectively) are inferior to the counterparts by HMA (1.93, 2.45, 2.48, and 3.22 mA cm^{-2} for Ta:Fe_2O_3, Ta:Fe_2O_3@Fe_2O_3, NiFe(OH)$_x$/Ta:Fe_2O_3, NiFe(OH)$_x$/Ta:Fe_2O_3@Fe_2O_3, respectively). The distinctive features of HMA are highly desirable for fabrication of high efficiency photoanodes, which cannot be achieved by CTA.

.

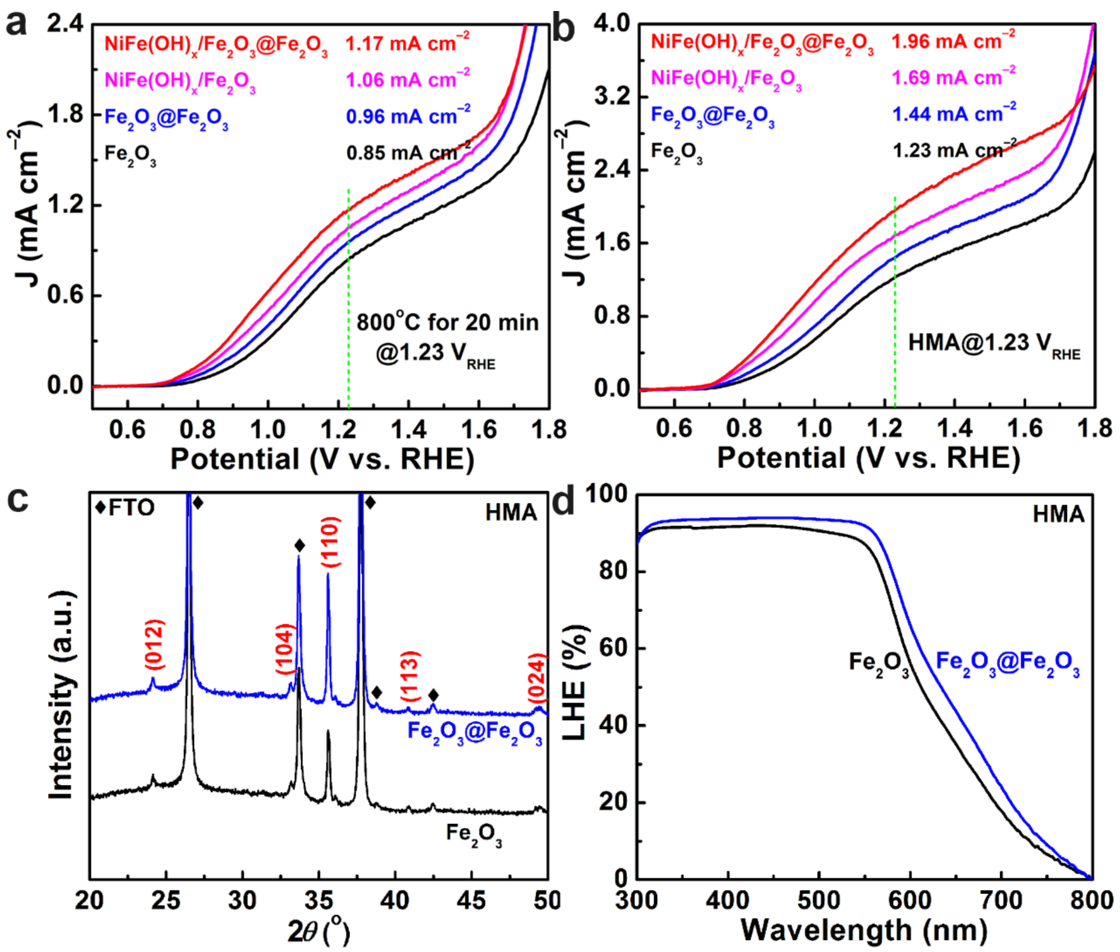

Supplementary Fig. 17| Bare hematite with/without second growth prepared by CTA and HMA. For both CTA (a) and HMA (b) samples, Fe_2O_3@Fe_2O_3 shows a limited increase of J_{ph} and a negligible V_{on} shift relative to bare hematite (J_{ph} is slightly better for HMA), which demonstrates that the built-in electric field does not occur in this homojunction. Therefore, the sufficient amount of dopants (M^{n+}, n>3) in the core part is essential to construct an effective homojunction, in which built-in electric field would promote charge separation efficiently. Note that $NiFe(OH)_x$ cocatalyst overlayer on all the HMA photoanodes promotes higher photocurrent density than on the CTA photoanodes, which demonstrates the high quality of electrodes prepared by HMA is reflected not only in their own behavior but also in the performance with cocatalyst loading. XRD (c) shows the similar pattern and intensity of peaks except the stronger intensity of (110) peak for homojunction, indicating the a preferential growth for bare hematite compared to Ta:Fe_2O_3. The LHE (d) of homojunction shows a slight increase relative to bare hematite, suggesting its negligible contribution to light absorption.

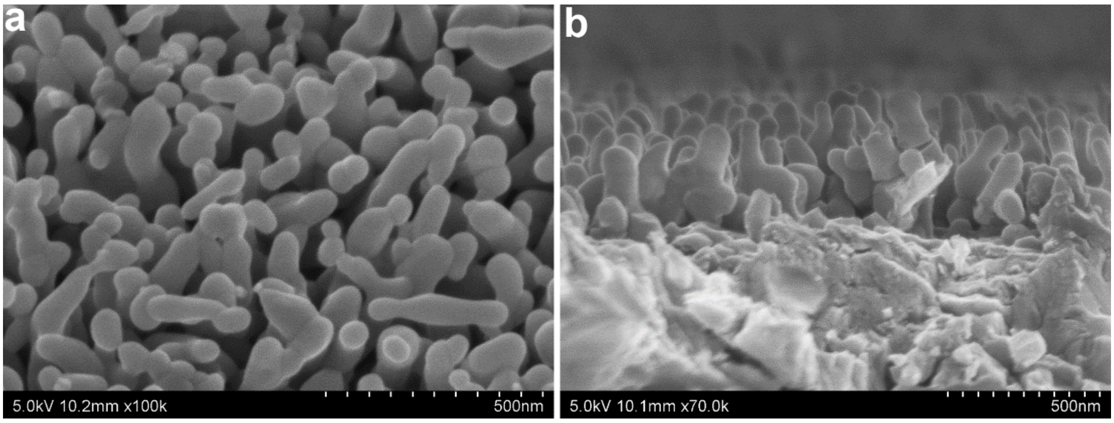

Supplementary Fig. 18| SEM images the CTA homojunction annealed at 800 ºC for 20 min. Clearly, the diameter of nanorods becomes bigger and nanorod tips turn blunt, which would affect surface area, conductivity, and light harvesting ability of nanorods, resulting in the poor performance. Moreover, the undesirable factors accompanying CTA also restrain the effect of $NiFe(OH)_x$ cocatalyst on the photoanodes, leading to a limited improvement of J_{ph}.

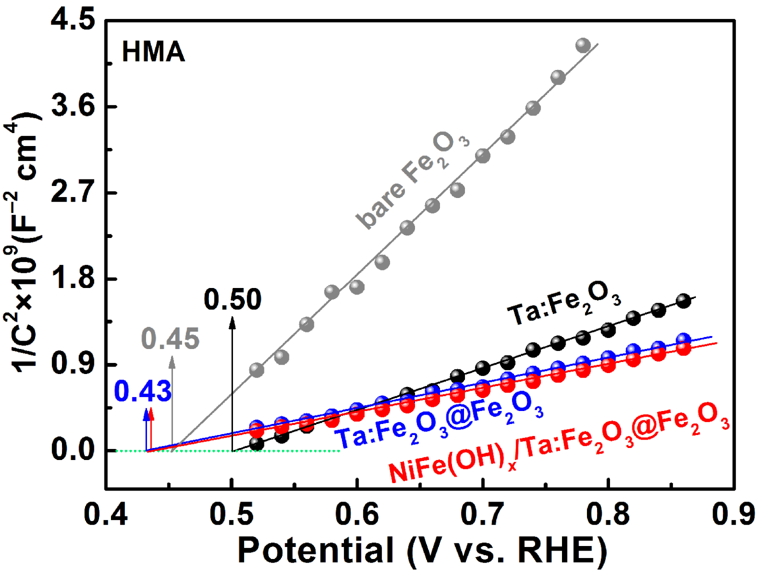

Supplementary Fig. 19| Mott–Schottky plots of bare Fe_2O_3, Ta:Fe_2O_3, Ta:Fe_2O_3@Fe_2O_3, and NiFe$(OH)_x$/Ta:Fe_2O_3@Fe_2O_3 photoanodes. Mott–Schottky plot can provide the flat band potential (E_{FB}, the intercept value on the X-axis), and the donor density (N_D, inversely proportional to the slope). Clearly, Ta:Fe_2O_3 shows a remarkably decreased slope compared to that of bare Fe_2O_3, indicating a significant increase of donor density to improve the poor conductivity of Fe_2O_3. On the other hand, E_{FB} of Ta:Fe_2O_3 shows a positive shift of ~50 mV, leading to unwanted increased overpotential (a side effect of doping). In stark contrast, homojunction formation creates a smaller slope and a lower E_{FB} relative to those of Ta:Fe_2O_3, which addresses both poor conductivity and high overpotential simultaneously, consistent with its higher photocurrent and lower V_{on}. Generally, the addition of an extremely thin cocatalyst layer should not affect the doping level or carrier density within hematite electrode. The presented N_D and E_{FB} of NiFe$(OH)_x$/Ta:Fe_2O_3@Fe_2O_3 are quite similar to those of homojunction, which are also in agreement with the previous reports of cocatalyst layer modification.[5, 6] It should be noted that Grätzel and coworkers have recently demonstrated through approximation of a flat surface and careful calculation of active surface area that an extremely thin cocatalyst layer ($CoFeO_x$) on hematite shifts only the photocurrent while leaving E_{FB} unchanged.[7, 8]

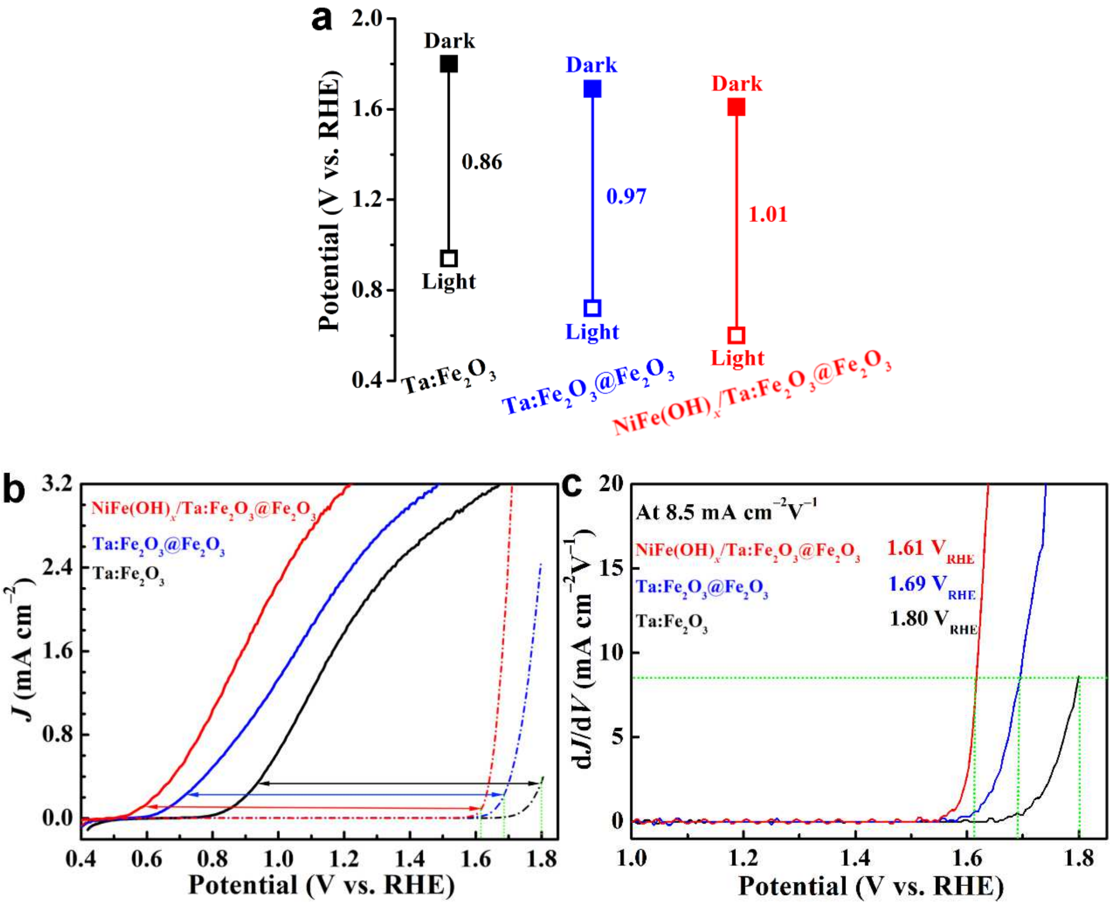

Supplementary Fig. 20| Extracted photovoltages. (**a**) The extracted photovoltage value of Ta:Fe_2O_3, Ta:Fe_2O_3@Fe_2O_3, and NiFe$(OH)_x$/Ta:Fe_2O_3@Fe_2O_3. (**b**) by the corresponding potential shifts between the dark and the light current density, and (**c**) by first-order derivatives of the corresponding dark voltammogram. Provided that the internal resistance of the photoanode is small (steep slope of the dark voltammogram), the photovoltage can be simply evaluated by the potential shift between the light and the dark current.[9] The first-order derivative of the dark voltammogram is also used to obtain the potential position, where the slope of the dark voltammogram is the same. In our case, all samples show very steep slope in the dark voltammogram at 8.5 mA $cm^{-2}V^{-1}$, which corresponds to the potential of 1.80, 1.69, and 1.61 V_{RHE} for Ta:Fe_2O_3, Ta:Fe_2O_3@Fe_2O_3, and NiFe$(OH)_x$/Ta:Fe_2O_3@Fe_2O_3, respectively.

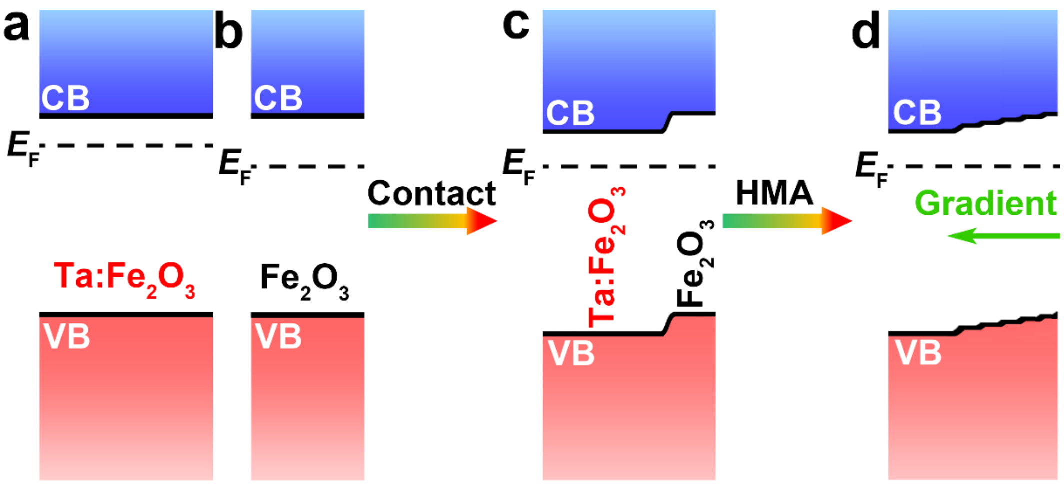

Supplementary Fig. 21| Schematic band diagram of Ta:Fe_2O_3@Fe_2O_3 homojunction. Compared with pure Fe_2O_3 (**b**), heavily doped Ta:Fe_2O_3 (**a**) shows a shifted Fermi level energy (E_F) closer to conduction band (CB). When they are brought into contact, however, E_F will equilibrate and produce additional band bending (**c**). Importantly, HMA would make a noticeable diffusion of Ta dopants from inside to outside, forming a gradient Ta doped hematite homojunction, which gives rise to a stepwise band bending (**d**) instead of steep one.

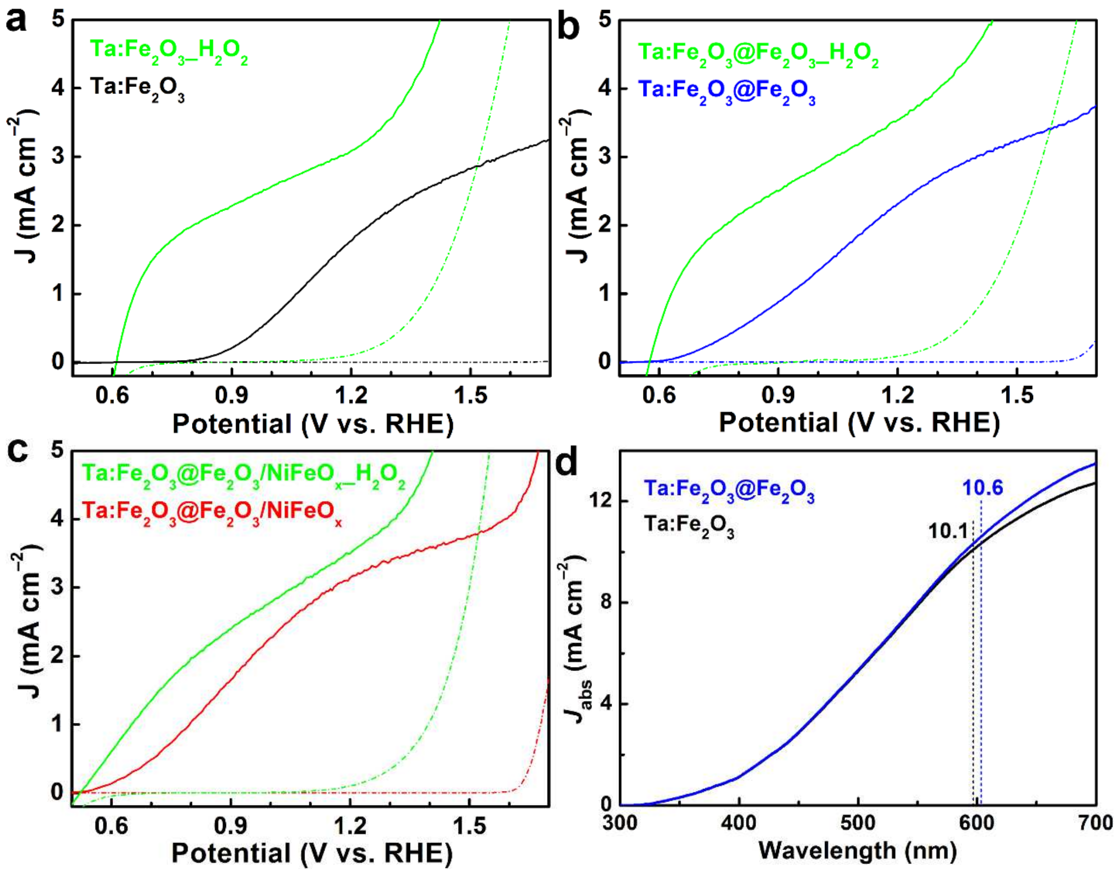

Supplementary Fig. 22| The measured *J*–*V* curves with and without addition of 0.5 M H_2O_2. (**a**) Ta:Fe_2O_3. (**b**) Ta:Fe_2O_3@Fe_2O_3. (**c**) NiFe$(OH)_x$/Ta:Fe_2O_3@Fe_2O_3. (**d**) Absorption photocurrents (J_{abs}) of Ta:Fe_2O_3 (10.1 mA cm^{-2}, <596 nm) and Ta:Fe_2O_3@Fe_2O_3 (10.6 mA cm^{-2}, <602 nm).

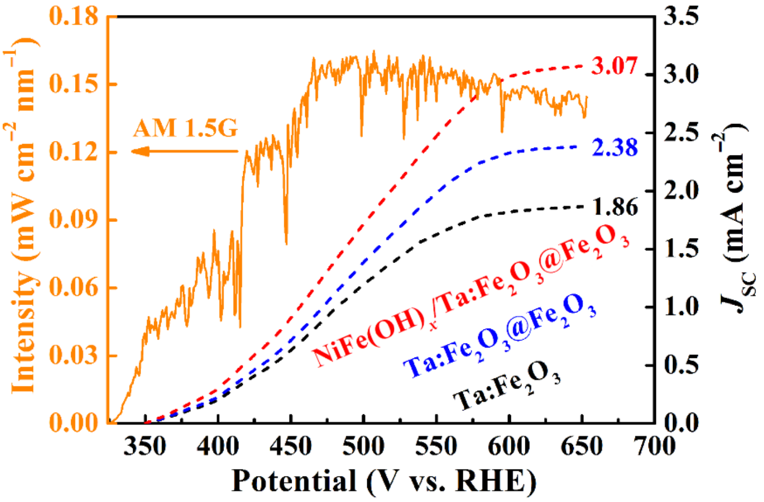

Supplementary Fig. 23| Integration of IPCE with respect to AM 1.5 G. The integrated photocurrent (J_{SC}) can be calculated using the following equation:

$$J_{SC} = \int_{350}^{650} \frac{1}{1240} \lambda \cdot IPCE(\lambda) \cdot E(\lambda) d(\lambda) \quad (8)$$

where $E(\lambda)$ is the solar irradiance at a specific wavelength (λ), $IPCE(\lambda)$ is the photoresponse profile of the photoanode at a specific wavelength (λ) at 1.23 V_{RHE}.

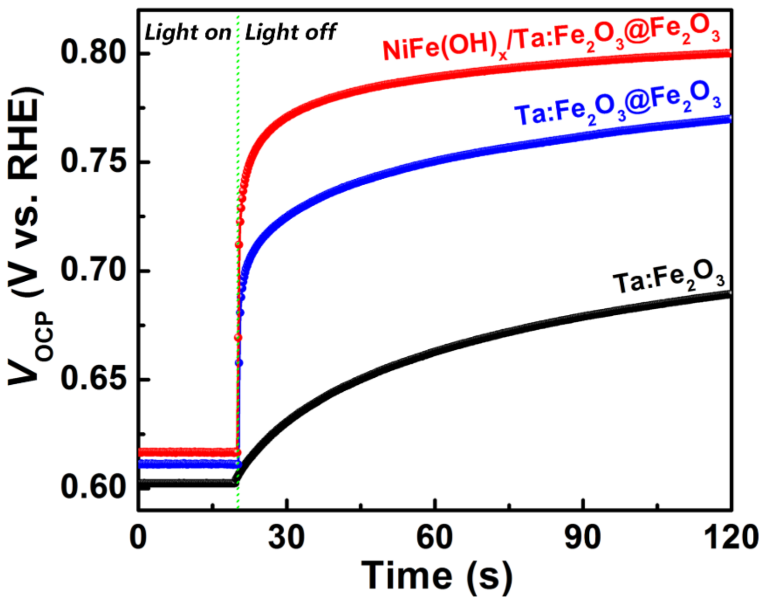

Supplementary Fig. 24| OCP transient decay profiles for Ta:Fe_2O_3, Ta:Fe_2O_3@Fe_2O_3, NiFe$(OH)_x$/Ta:Fe_2O_3@Fe_2O_3. The OCP transient decay evaluates the surface recombination between trapped electrons and reaction intermediates rather than the bulk recombination and this process is very fast (within ns–ms domain), while the scale of OCP decay is usually in several minutes.[10] Generally, OCP is very positive in dark due to a largely upward band bending, while OCP will be more cathodic under illumination due to flattening of the energy band by photoexcited carriers. ΔOCP (OCP_{dark} – OCP_{light}), also known as photovoltage, represents the amount of band bending under illumination with respect to that in the dark condition. Homojunction formation boosts ΔOCP value compared to that of Ta:Fe_2O_3, indicating that homojunction can generate an additional built-in electric field. At the transient time from illumination (quasi-equilibrium of flattened energy band) to the dark (equilibrium of bent energy band), the charge recombination strongly depends on the spatial charges built in the photoanode/electrolyte junction. The strong band bending enables a large amount of spatial charges in the depletion region and thus significant charge recombination occurs at the transient when the illumination is removed. As such, a fast OCP-decay is expected.

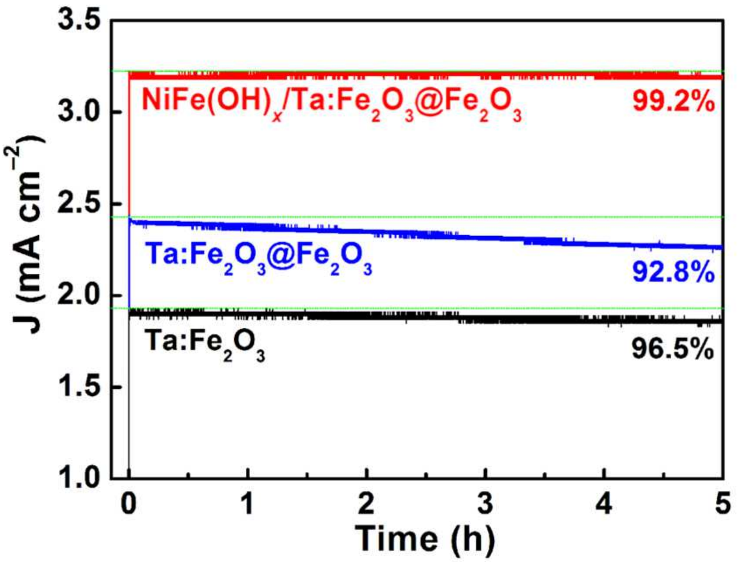

Supplementary Fig. 25| Stability measurement. All samples show excellent stability tests in 5 h (all remain more than 90%). The stability order is NiFe(OH)$_x$/Ta:Fe_2O_3@Fe_2O_3 (99.2%) > Ta:Fe_2O_3 (96.5%) > Ta:Fe_2O_3@Fe_2O_3 (92.8%).

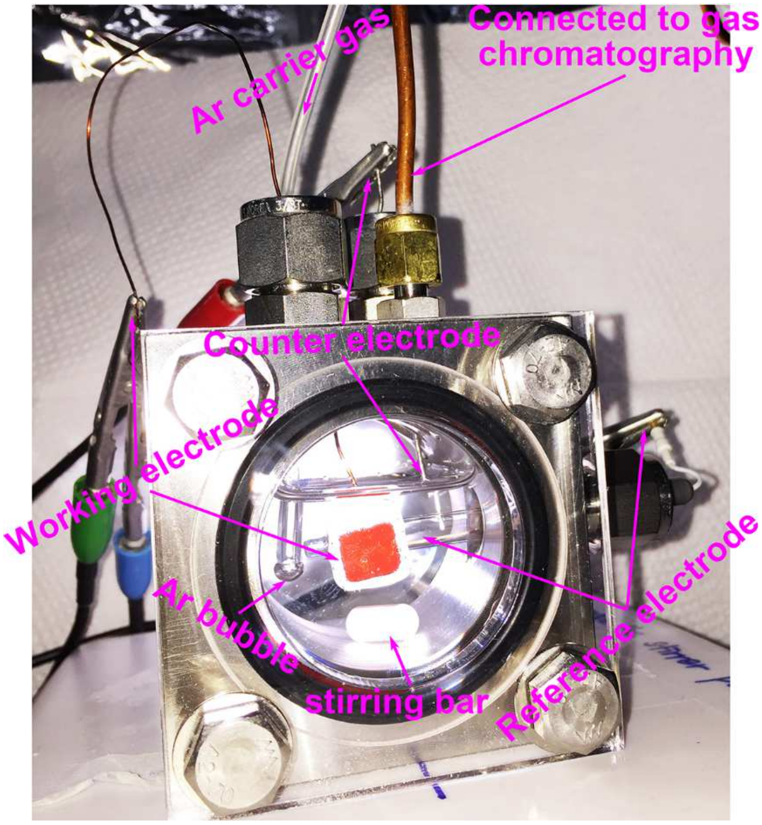

Supplementary Fig. 26| A typical PEC system used in this study with a closed circulation for the measurement of H_2 and O_2 evolution.

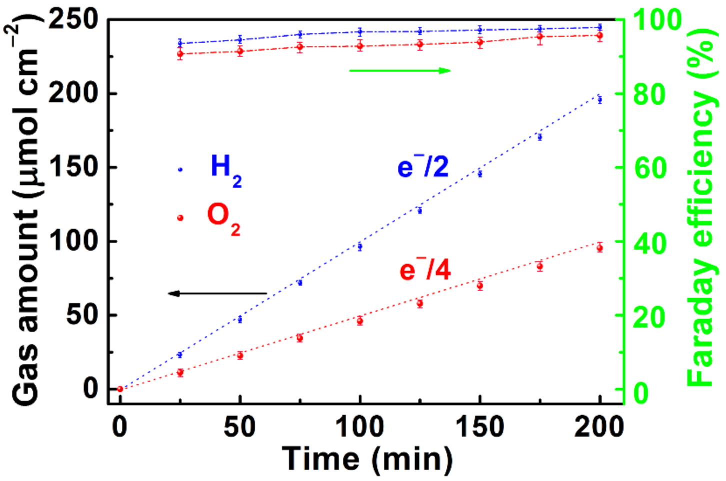

Supplementary Fig. 27| Evolution of O_2 and H_2 over NiFe(OH)$_x$/Ta:Fe_2O_3@Fe_2O_3 photoanode at a constant potential of 1.23 V_{RHE}. Each point represents an average value from three sets of measurements. The ratio of evolved O_2 and H_2 is close to stoichiometry, and F.E.'s of O_2 and H_2 evolution reaction is 92.3 % and 95.8 %, respectively. The dashed lines are theoretical amount of O_2 and H_2 evolution at 1.23 V_{RHE} (3.22 mA cm^{-2}) under simulated 1 sun irradiation. Faraday efficiency (F.E.) was calculated by following equation:

$$\text{F.E. (\%)} = \frac{moles\ \ of\ \ the\ \ product\ \ actually\ \ measured}{theoretical\ \ moles\ \ based\ \ on\ \ measured\ \ current} \times 100\ \ \% \qquad (9)$$

The ratio of evolved O_2 and H_2 is close to stoichiometry, and F.E.'s of O_2 and H_2 evolution reaction is 92.3 % and 95.8 %, respectively.

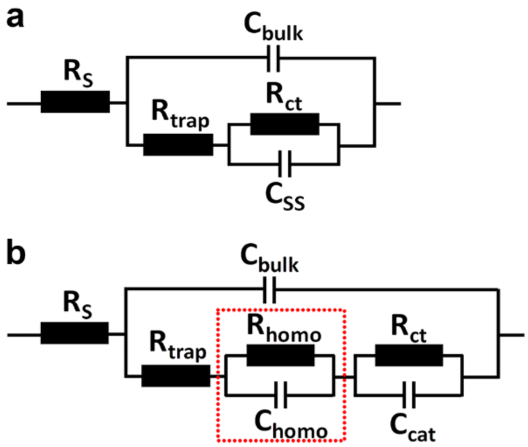

Supplementary Fig. 28| Representative two- and three-RC-unit equivalent fitting models. A typical two-RC-unit equivalent circuit generally consists of three resistances and two capacitors: a series resistance (R_s, essentially small and constant) by the electrolyte, external contact and conductive substrate layer, a trapping resistance (R_{trap}) at surface states by the trapping holes, a charge transfer resistance (R_{ct}) at semiconductor–liquid junction, a bulk capacitor of space charge region (C_{bulk}), and a surface states capacitor (C_{ss}). Three-RC-unit equivalent circuit is used to fit the cocatalyst-modified homojunction beyond 1.1 V_{RHE} where a third circle shows up clearly (see Supplementary Fig. 28c).

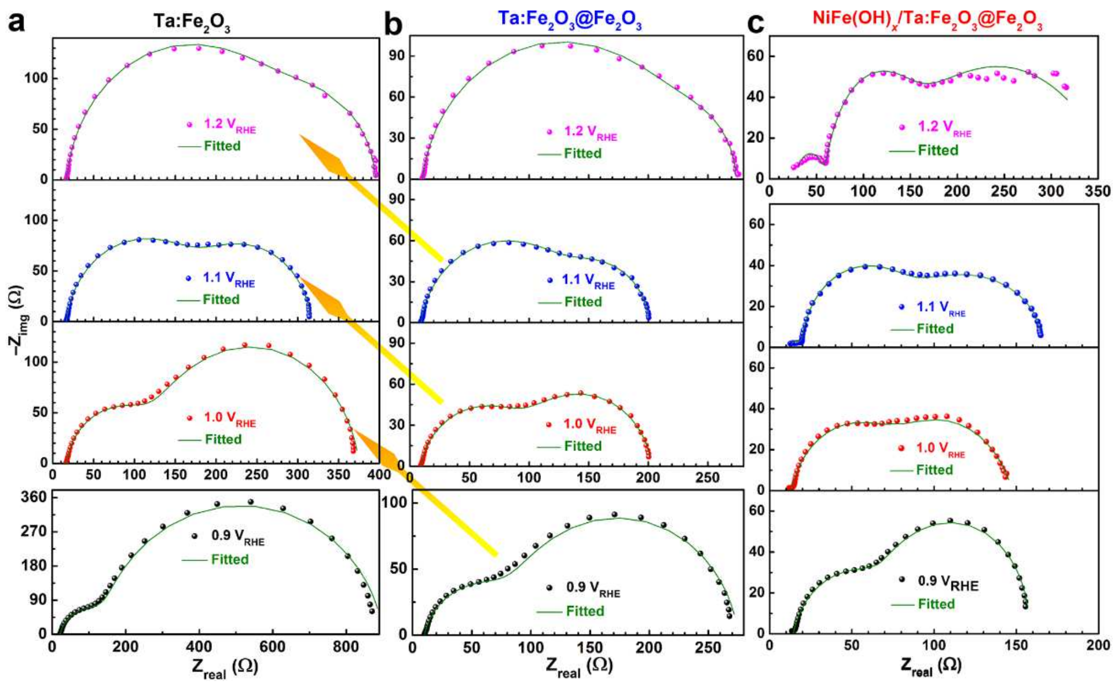

Supplementary Fig. 29| Nyquist plots at different potentials. Scattered dots are measured data, solid lines are fitting results. **(a)** Ta:Fe_2O_3. **(b)** Ta:Fe_2O_3@Fe_2O_3. **(c)** NiFe(OH)$_x$/Ta:Fe_2O_3@Fe_2O_3. All PEIS spectra of our three samples have a good fitting to the equivalent circuits and no other constant phases are required. Compared with Ta:Fe_2O_3, homojunction shows an almost same shape of Nyquist plot at earlier potential (at least 100 mV in advance, see highlighting arrows), which is consistent with the measured V_{on}. At the same potential, homojunction shows the smaller total resistance than that of Ta:Fe_2O_3, confirming the beneficial effect of charge separation. By cocatalyst modification, the total resistance is further reduced (< 1.1 V_{RHE}) by greatly improved surface charge transfer.

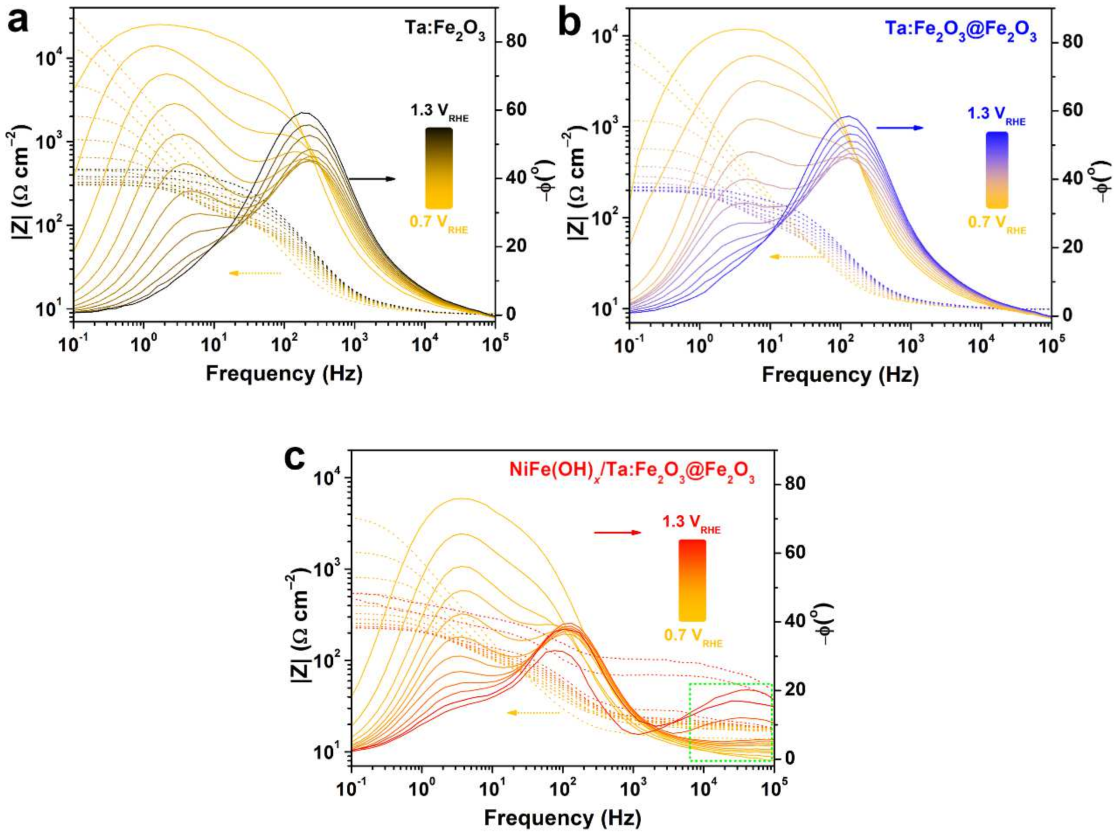

Supplementary Fig. 30| Bode plots of Ta:Fe_2O_3 (a), Ta:Fe_2O_3@Fe_2O_3 (b), and NiFe$(OH)_x$/Ta:Fe_2O_3@Fe_2O_3 (c). Yellow lines represent lower potentials. Both Ta:Fe_2O_3 and Ta:Fe_2O_3@Fe_2O_3 show near zero phase angles at frequencies between 10^4 and 10^5 Hz, which suggests almost no capacitance at electrode/sample interface. In contrast, NiFe$(OH)_x$/Ta:Fe_2O_3@Fe_2O_3 shows significantly increasing phase angles at this region (green dotted circle), indicating the appearance of new capacitance.

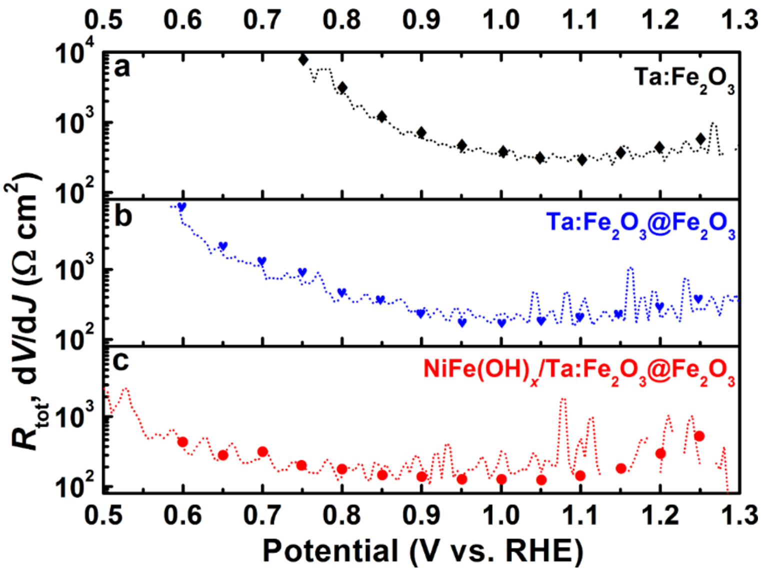

Supplementary Fig. 31| PEIS fitted total resistance (*R*$_{tot}$, scattered points**) and differential resistances (*R’*$_{tot}$,** dotted lines**). (a)** Ta:Fe_2O_3, **(b)** Ta:Fe_2O_3@Fe_2O_3, and **(c)** NiFe(OH)$_x$/Ta:Fe_2O_3@Fe_2O_3.

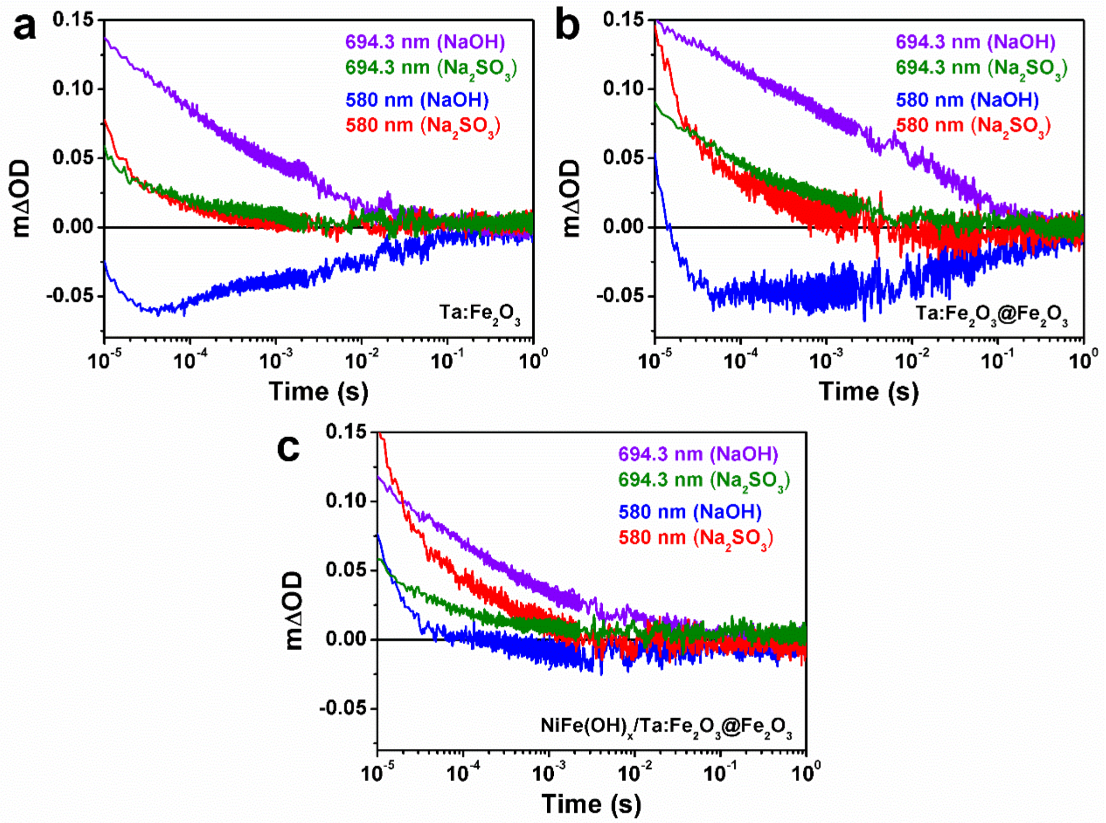

Supplementary Fig. 32| Transient absorption spectroscopy (TAS) for Ta:Fe_2O_3, Ta:Fe_2O_3@Fe_2O_3, and NiFe(OH)$_x$/Ta:Fe_2O_3@Fe_2O_3 photoanodes in electrolyte of 1 M NaOH with or without 0.5 M Na_2SO_3.

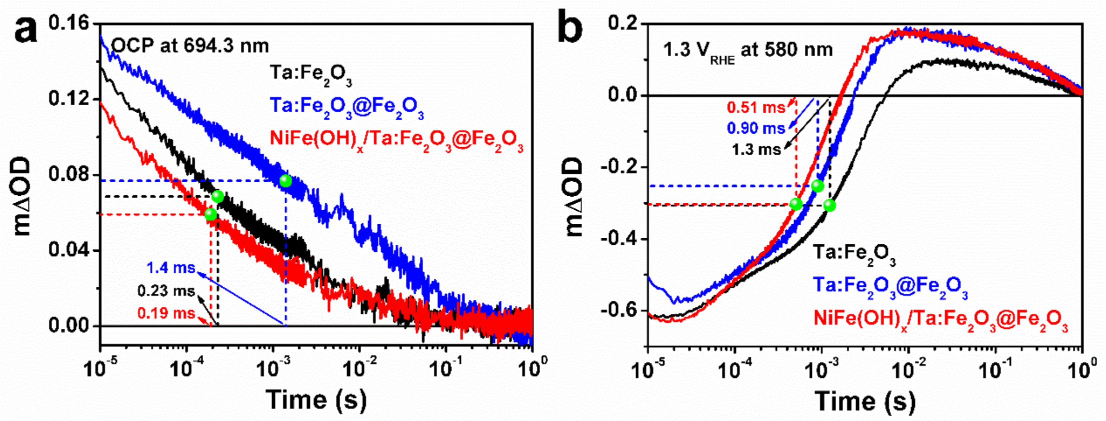

Supplementary Fig. 33| TAS and corresponding half lifetime ($\tau_{50\%}$) for $Ta:Fe_2O_3$, $Ta:Fe_2O_3@Fe_2O_3$ and $NiFe(OH)_x/Ta:Fe_2O_3@Fe_2O_3$ photoanodes. (a) OCP at 694.3 nm. (b) 1.3 V_{RHE} at 580 nm. $\tau_{50\%}$ is the time when the TAS amplitude decreases by half of its initial value at 10 μs.[11]

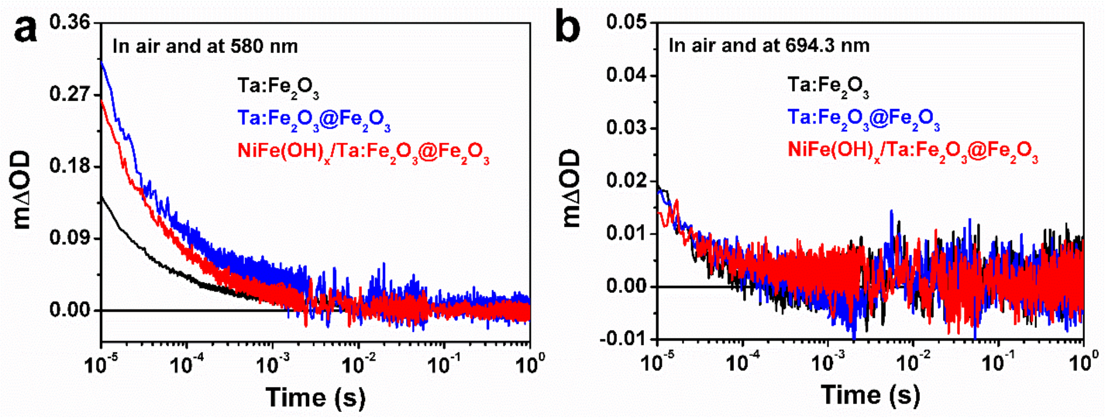

Supplementary Fig. 34| TAS for $Ta:Fe_2O_3$, $Ta:Fe_2O_3@Fe_2O_3$, and $NiFe(OH)_x/Ta:Fe_2O_3@Fe_2O_3$ photoanodes in air. In air, three samples show negligible absorption at 694.3 nm and fast decay at 580 nm. The order of amplitude at 10 μs is as follows: $Ta:Fe_2O_3@Fe_2O_3$ > $NiFe(OH)_x/Ta:Fe_2O_3@Fe_2O_3$ > $Ta:Fe_2O_3$. It demonstrates that Ta doping and homojunction fabrication can promote the formation of long-lived photoinduced charges.

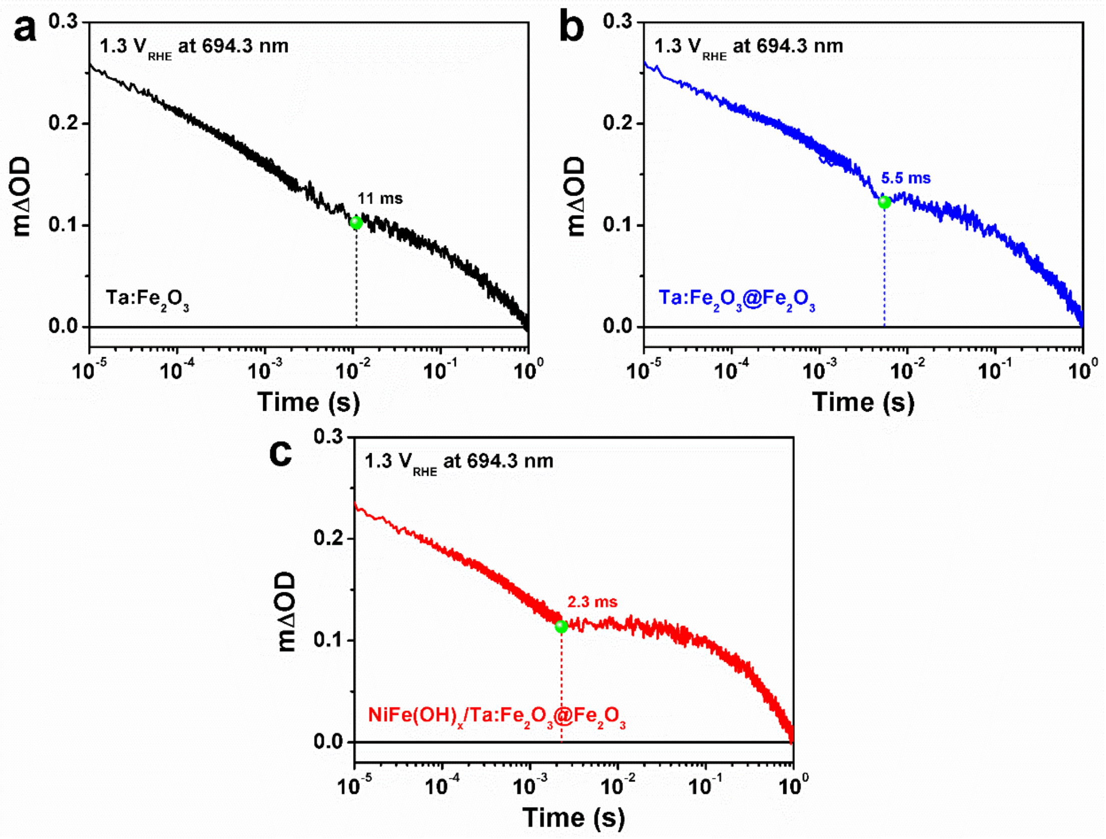

Supplementary Fig. 35| The turning point of biphasic decay at 694.3 nm and 1.3 V_{RHE} for Ta:Fe_2O_3 (**a**), Ta:Fe_2O_3@Fe_2O_3 (**b**) and NiFe$(OH)_x$/Ta:Fe_2O_3@Fe_2O_3 (**c**) photoanodes. At 1.3 V_{RHE}, the decay dynamics at 694.3 nm displays biphasic decay, including recombination process at μs–ms and water oxidation process at ms–s.[12] To compare the timescale of starting water oxidation, the turning point is extracted from biphasic decay curve. The shorter the turning point time is, the more photoholes the water oxidation employs effectively.

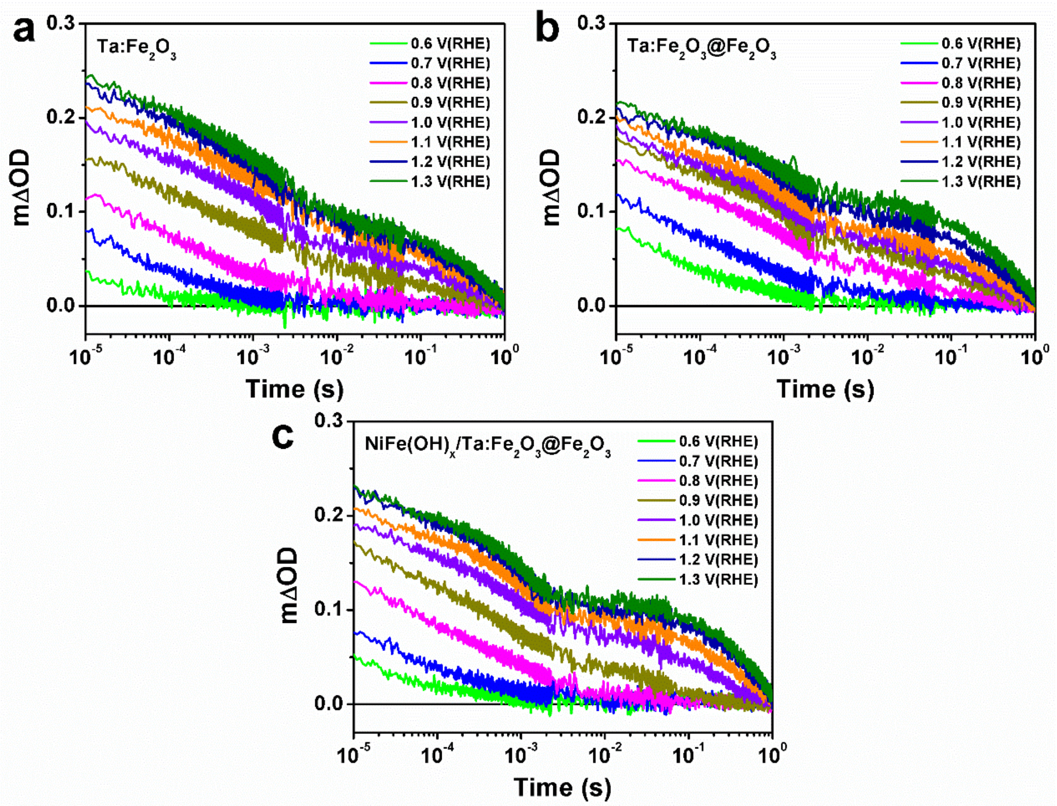

Supplementary Fig. 36| Bias-dependent TAS for Ta:Fe_2O_3, Ta:Fe_2O_3@Fe_2O_3, and NiFe$(OH)_x$/Ta:Fe_2O_3@Fe_2O_3 photoanodes in 1M NaOH. Bias-dependent TAS results display that the long-lived holes appear at lower bias (0.7–0.8 V_{RHE}) in homojunction photoanode than that of the Ta:Fe_2O_3, in accordance with the negative shift of onset potential. However, no long-lived holes were observed at lower bias (0.7–0.8 V_{RHE}) after NiFe$(OH)_x$ modification. This result may be related to two reasons. One is the hole transfer from homojunction to NiFe$(OH)_x$. The other is the hole absorption spectra in NiFeOOH overlapped with the photoholes in Fe_2O_3 (the reactive center in NiFeOOH electrocatalyst is Fe-site), which cannot be distinguished from the hole in hematite.[13] Accordingly, at applied bias of 1.3 V_{RHE} (Fig. 6d), Ta:Fe_2O_3@Fe_2O_3/NiFe$(OH)_x$ photoanode shows similar dynamics with Ta:Fe_2O_3@Fe_2O_3 at ms-s timescale probably due to the similar absorption coefficient of NiFe$(OH)_x$ with Fe_2O_3.[14]

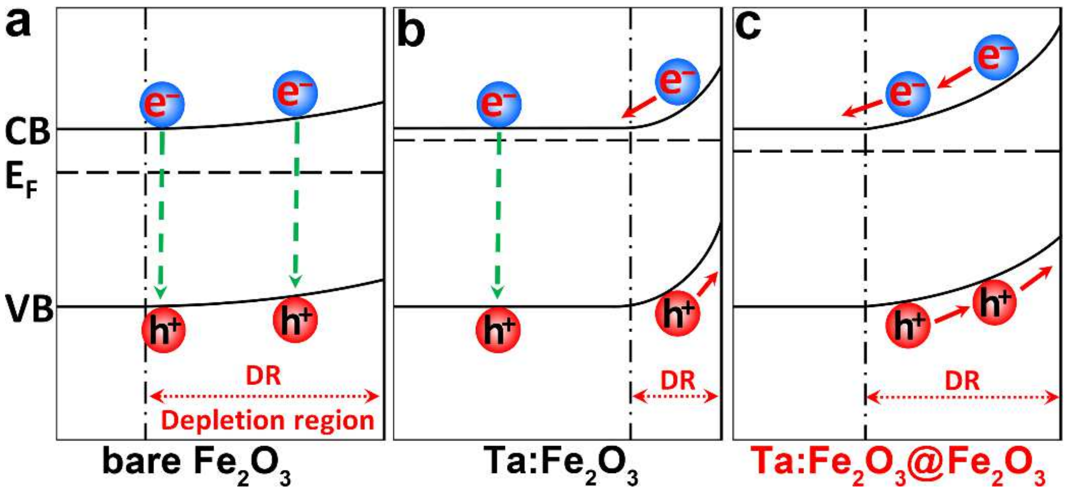

Supplementary Fig. 37| Variation comparison of deletion region between bare Fe_2O_3, Ta:Fe_2O_3, and Ta:Fe_2O_3@Fe_2O_3.

Supplementary Table 1. Recently reported doped-hematite photoanodes with/without cocatalyst.

**Doped hematite photoanode /with cocatalyst**	**Photocurrent density (1.23 V_{RHE})**	**Reference**
Si:Fe_2O_3<br>/with Co-Pi	2.20 mA cm^{-2}<br>2.70 mA cm^{-2}	[15]*JACS*,<br>**128**, 15714 (2006).
Pt:Fe_2O_3<br>/with Co-Pi	2.19 mA cm^{-2}<br>4.32 mA cm^{-2}	[16]*Sci Rep*,<br>**3**, 2681 (2013).
Ge:Fe_2O_3	2.19 mA cm^{-2}	[17]*Nano Energy*,<br>**9**, 282 (2014).
Ti:Fe_2O_3<br>/with $CoFeO_x$	0.92 mA cm^{-2}<br>2.49 mA cm^{-2}	[18]*Adv Funct Mater*,<br>**26**, 4414 (2016).
Ti:Fe_2O_3<br>/with Ti-SiO_x/Co-Pi	1.25 mA cm^{-2}<br>3.19 mA cm^{-2}	[19]*Angew Chem Int Ed*,<br>**55**, 9922 (2016).
Sn, Be:Fe_2O_3	1.70 mA cm^{-2}	[20]*Sci Rep*,<br>**6**, 23183 (2016).
P:Fe_2O_3<br>/with Co-Pi	1.48 mA cm^{-2}<br>2.00 mA cm^{-2}	[21]*Chem Sci*,<br>**8**, 91 (2017).
Sn:Fe_2O_3<br>/with cobalt oxide	1.38 mA cm^{-2}<br>2.20 mA cm^{-2}	[22]*Nano Letters*,<br>**17**, 2490 (2017).
Zr:Fe_2O_3<br>/with Co-Pi	1.38 mA cm^{-2}<br>2.20 mA cm^{-2}	[23]*Angew Chem Int Ed*,<br>**56**, 4150 (2017).
P:Fe_2O_3<br>/with Mg-Fe_2O_3	1.30 mA cm^{-2}<br>2.40 mA cm^{-2}	[24]*J Mater Chem A*,<br>**6**, 13412 (2018).
Nb, Sn:Fe_2O_3<br>/with activation	1.88 mA cm^{-2}<br>3.05 mA cm^{-2}	[25]*Chem Sci*,<br>**10**, 10436 (2019).
**Ta:Fe_2O_3**<br>**/with NiFe$(OH)_x$**	**1.93 mA cm^{-2}**<br>**2.48 mA cm^{-2}**	**This work**

Supplementary Table 2. Performance of recently-reported hematite-based photoanodes.

**Hematite-based photoanodes /with cocatalyst**	**Photocurrent density (1.23 V_{RHE})**	**Onset potential (V_{on})**	**Reference**
Dual-regrowth Fe_2O_3 /with $NiFeO_x$	1.15 mA cm^{-2} 1.30 mA cm^{-2}	0.67 V_{RHE} 0.45 V_{RHE}	[26]*Nat Commun*, **6**, 7447 (2015).
Fe_2O_3@Fe_2TiO_5 /with Co-Pi	2.00 mA cm^{-2} 2.60 mA cm^{-2}	0.96 V_{RHE} 0.90 V_{RHE}	[27]*ACS Nano*, **9**, 5348 (2015).
SnO_2-SiO_x/Ti:Fe_2O_3 /with FeOOH	1.25 mA cm^{-2} 1.54 mA cm^{-2}	0.85 V_{RHE} 0.64 V_{RHE}	[28]*Adv Energy Mater*, **6**, 1501840 (2016).
Fe_2O_3@Fe_2TiO_5 /with $FeNiO_x$	1.63 mA cm^{-2} 2.70 mA cm^{-2}	0.95 V_{RHE} 0.80 V_{RHE}	[29]*Small*, **12**, 3415 (2016).
Fe_2O_3/TiO_2 / with $Fe_{20}Cr_{40}Ni_{40}O_x$	1.90 mA cm^{-2} 2.50 mA cm^{-2}	0.90 V_{RHE} 0.8 V_{RHE}	[30]*ACS Omega*, **2**, 4531 (2017).
ITO/Fe_2O_3/Fe_2TiO_5 / with FeNiOOH	1.56 mA cm^{-2} 2.20 mA cm^{-2}	1.00 V_{RHE} 0.95 V_{RHE}	[31]*Energy Environ Sci*, **10**, 2124 (2017).
Ti,Sn:Fe_2O_3/B:Fe_2O_3 /with FeOOH	1.92 mA cm^{-2} 2.35 mA cm^{-2}	0.92 V_{RHE} 0.84 V_{RHE}	[32]*ACS Catal*, **8**, 11932 (2018).
Fe_2O_3 /with F:FeOOH/FeNiOOH	0.83 mA cm^{-2} 1.05 mA cm^{-2}	0.85 V_{RHE} 0.55 V_{RHE}	[33]*ChemsusChem*, **11**, 3783 (2018).
Ti:Fe_2O_3 mesocrystal /with Co-Pi	2.50 mA cm^{-2} 3.50 mA cm^{-2}	0.90 V_{RHE} 0.86 V_{RHE}	[34]*Nat Commun*, **10**, 4832 (2019).
Ti:Fe_2O_3/Co_3O_4 /with Co-Pi	2.00 mA cm^{-2} 2.70 mA cm^{-2}	0.64 V_{RHE} 0.64 V_{RHE}	[10]*Adv Funct Mater*, **29**, 1801902 (2019).
Nb,Sn:Fe_2O_3@$FeNbO_4$ /with $NiFeO_x$	2.24 mA cm^{-2} 2.71 mA cm^{-2}	0.80 V_{RHE} 0.71 V_{RHE}	[4]*ACS Catal*, **9**, 1289 (2019).
H_2 treated Fe_2O_3/TiO_2 /with Co-Pi	4.5 mA cm^{-2} 6.0 mA cm^{-2}	0.78 V_{RHE} 0.58 V_{RHE}	[35]*Nano Energy*, **39**, 211 (2017).
Fe_2O_3/Al_2O_3 /with $CoFeO_x$	1.60 mA cm^{-2} 2.50 mA cm^{-2}	1.00 V_{RHE} 0.80 V_{RHE}	[8]*J Mater Chem A*, **7**, 6012 (2019).
**Ta:Fe_2O_3@Fe_2O_3 homojunction /with $NiFe(OH)_x$**	**2.45 mA cm^{-2} 3.22 mA cm^{-2}**	**0.63 V_{RHE} 0.55 V_{RHE}**	**This work**

Note: To have a consistent comparison, the onset potentials of photoanodes in all references were reevaluated by extrapolation-interception method.

Supplementary References

1. Formal FL, Grätzel M, Sivula K. Controlling photoactivity in ultrathin hematite films for solar water‐splitting. *Adv Funct Mater* **20**, 1099-1107 (2010).

2. Du C, *et al.* Hematite-based water splitting with low turn-on voltages. *Angew Chem Int Ed* **52**, 12692-12695 (2013).

3. Yu Q, Meng X, Wang T, Li P, Ye J. Hematite films decorated with nanostructured ferric oxyhydroxide as photoanodes for efficient and stable photoelectrochemical water splitting. *Adv Funct Mater* **25**, 2686-2692 (2015).

4. Zhang H, Kim YK, Jeong HY, Lee JS. A few atomic $FeNbO_4$ overlayers on hematite nanorods: microwave-induced high temperature phase for efficient photoelectrochemical water splitting. *ACS Catal* **9**, 1289-1297 (2019).

5. Li M, *et al.* Zipping up $NiFe(OH)_x$-encapsulated hematite to achieve an ultralow turn-on potential for water oxidation. *ACS Energy Lett* **4**, 1983-1990 (2019).

6. Malara F, *et al.* α-Fe_2O_3/NiOOH: an effective heterostructure for photoelectrochemical water oxidation. *ACS Catal* **5**, 5292-5300 (2015).

7. Cesar I, Sivula K, Kay A, Zboril R, Grätzel M. Influence of feature size, film thickness, and silicon doping on the performance of nanostructured hematite photoanodes for solar water splitting. *J Phys Chem C* **113**, 772-782 (2009).

8. Liardet L, Katz JE, Luo J, Grätzel M, Hu X. An ultrathin cobalt–iron oxide catalyst for water oxidation on nanostructured hematite photoanodes. *J Mater Chem A* **7**, 6012-6020 (2019).

9. Dotan H, Mathews N, Hisatomi T, Grätzel M, Rothschild A. On the solar to hydrogen conversion efficiency of photoelectrodes for water splitting. *J Phys Chem Lett* **5**, 3330-3334 (2014).

10. Yi S-S, Wulan B-R, Yan J-M, Jiang Q. Highly efficient photoelectrochemical water splitting: surface modification of cobalt-phosphate-loaded Co_3O_4/Fe_2O_3 p–n heterojunction nanorod arrays. *Adv Funct Mater* **29**, 1801902 (2019).

11. Jing L, Zhou J, Durrant JR, Tang J, Liu D, Fu H. Dynamics of photogenerated charges in the phosphate modified TiO_2 and the enhanced activity for photoelectrochemical water splitting. *Energy Environ Sci* **5**, 6552-6558 (2012).

12. Pendlebury SR, *et al.* Correlating long-lived photogenerated hole populations with photocurrent densities in hematite water oxidation photoanodes. *Energy Environ Sci* **5**, 6304-6312 (2012).

13. Chung DY, *et al.* Dynamic stability of active sites in hydr(oxy)oxides for the oxygen evolution reaction. *Nat Energy* **5**, 222-230 (2020).

14. Francàs L, *et al.* Spectroelectrochemical study of water oxidation on nickel and iron oxyhydroxide electrocatalysts. *Nat Commun* **10**, 5208 (2019).

15. Kay A, Cesar I, Grätzel M. New benchmark for water photooxidation by nanostructured α-

Fe_2O_3 films. *J Am Chem Soc* **128**, 15714-15721 (2006).

16. Kim JY, *et al.* Single-crystalline, wormlike hematite photoanodes for efficient solar water splitting. *Sci Rep* **3**, 2681 (2013).
17. Liu J, *et al.* Highly oriented Ge-doped hematite nanosheet arrays for photoelectrochemical water oxidation. *Nano Energy* **9**, 282-290 (2014).
18. Xu Y-F, Wang X-D, Chen H-Y, Kuang D-B, Su C-Y. Toward high performance photoelectrochemical water oxidation: combined effects of ultrafine cobalt iron oxide nanoparticle. *Adv Funct Mater* **26**, 4414-4421 (2016).
19. Ahn H-J, Yoon K-Y, Kwak M-J, Jang J-H. A titanium-doped SiO_x passivation layer for greatly enhanced performance of a hematite-based photoelectrochemical system. *Angew Chem Int Ed* **55**, 9922-9926 (2016).
20. Annamalai A, *et al.* Sn/Be sequentially co-doped hematite photoanodes for enhanced photoelectrochemical water oxidation: effect of Be^{2+} as co-dopant. *Sci Rep* **6**, 23183 (2016).
21. Luo Z, Li C, Liu S, Wang T, Gong J. Gradient doping of phosphorus in Fe_2O_3 nanoarray photoanodes for enhanced charge separation. *Chem Sci* **8**, 91-100 (2017).
22. Li M, *et al.* Morphology and doping engineering of Sn-doped hematite nanowire photoanodes. *Nano Lett* **17**, 2490-2495 (2017).
23. Li C, *et al.* Surviving high-temperature calcination: ZrO_2-induced hematite nanotubes for photoelectrochemical water oxidation. *Angew Chem Int Ed* **56**, 4150-4155 (2017).
24. Li F, *et al.* Facile regrowth of Mg-Fe_2O_3/P-Fe_2O_3 homojunction photoelectrode for efficient solar water oxidation. *J Mater Chem A* **6**, 13412-13418 (2018).
25. Zhang H, Park JH, Byun WJ, Song MH, Lee JS. Activating the surface and bulk of hematite photoanodes to improve solar water splitting. *Chem Sci* **10**, 10436-10444 (2019).
26. Jang JW, *et al.* Enabling unassisted solar water splitting by iron oxide and silicon. *Nat Commun* **6**, 7447 (2015).
27. Deng J, *et al.* Thin-layer Fe_2TiO_5 on hematite for efficient solar water oxidation. *ACS Nano* **9**, 5348-5356 (2015).
28. Cho IS, Han HS, Logar M, Park J, Zheng X. Enhancing low-bias performance of hematite photoanodes for solar water splitting by simultaneous reduction of bulk, interface, and surface recombination pathways. *Adv Energy Mater* **6**, 1501840 (2016).
29. Li C, Wang T, Luo Z, Liu S, Gong J. Enhanced charge separation through ALD-modified Fe_2O_3/Fe_2TiO_5 nanorod heterojunction for photoelectrochemical water oxidation. *Small* **12**, 3415-3422 (2016).
30. Bärtsch M, *et al.* Multicomposite nanostructured hematite–titania photoanodes with improved oxygen evolution: the role of the oxygen evolution catalyst. *ACS Omega* **2**, 4531-4539 (2017).
31. Tang P, *et al.* Enhanced photoelectrochemical water splitting of hematite multilayer nanowires photoanode with tuning surface state via bottom-up interfacial engineering.

Energy Environ Sci **10**, 2124-2136 (2017).

32. Ahn H-J, Yoon K-Y, Kwak M-J, Park J, Jang J-H. Boron-doping of metal-doped hematite for reduced surface recombination in water splitting. *ACS Catal* **8**, 11932-11939 (2018).
33. Deng J, *et al.* Efficient photoelectrochemical water oxidation on hematite with fluorine-doped FeOOH and FeNiOOH as dual cocatalysts. *ChemSusChem* **11**, 3783-3789 (2018).
34. Zhang Z, *et al.* Interfacial oxygen vacancies yielding long-lived holes in hematite mesocrystal-based photoanodes. *Nat Commun* **10**, 4832 (2019).
35. Jeon TH, Moon G-h, Park H, Choi W. Ultra-efficient and durable photoelectrochemical water oxidation using elaborately designed hematite nanorod arrays. *Nano Energy* **39**, 211-218 (2017).

Supplementary Note Section:

The obtained photocurrent density, J_{H_2O}, could be represented as following:

$$J_{H_2O} = J_{abs} \times \eta_{bulk} \times \eta_{surface} \quad (1)$$

Since there is no injection barrier of holes for H_2O_2 oxidation ($\eta_{surface} = 1$)

$$J_{H_2O_2} = J_{abs} \times \eta_{bulk} \quad (2)$$

Hence $\eta_{surface} = J_{H_2O}/J_{H_2O_2}$ (3)

And $\eta_{bulk} = J_{H_2O_2}/J_{abs}$ (4)

Here, $J_{H_2O_2}$ is the photocurrent density measured in 1 M KOH with 0.5 M H_2O_2 and J_{abs} is the expected photocurrent density when absorbed photons are completely converted into current. For calculation of J_{abs}, the correlation between absorbance and irradiation is the following:

$$P_d = P_0 10^{-A} \quad (5)$$

$$P_{abs} = P_0(1 - 10^{-A}) \quad (6)$$

P_0 (unit: mW cm^{-2} nm^{-1}) is power provided by solar simulator (in this case, AM 1.5G), P_{abs} is power of light actually absorbed by photoanode and P_d is power of light not absorbed at the photoanode but dissipated by reflection and penetration. A is absorbance of photoanode and LHE (light harvesting efficiency) is defined as $1 - 10^{-A}$. So light not absorbed at the photoanode will be 10^{-A}. Integration of $P_{abs}(\lambda)$ (mW cm^{-2} nm^{-1}) along with wavelength λ gives total power density (unit of mW cm^{-2}), which is the power of light absorbed by photoanode (maximum power of photoanode). The following formula shows such relationship photon absorption (J_{abs}):

$$J_{abs} = \int_{\lambda_1}^{\lambda_2} \frac{\lambda}{1240} P_{abs(\lambda)}\, d\lambda \quad \left(\frac{mW}{cm^2}\right) \quad (7)$$
